# Supplementary material for: A strategy for Cas13 miniaturization based on the structure and AlphaFold
Source: Nat Commun. 2023 Sep 8;14:5545. doi: 10.1038/s41467-023-41320-8 (PMC10491665; doi:10.1038/s41467-023-41320-8)
Supplement: Supplementary file 1 — Supplementary Information [file 41467_2023_41320_MOESM1_ESM.pdf]

Supplementary Information

Supplementary Figure 1. The workflow of IDC strategy.

Supplementary Figure 2. Western blot assay of Cas13 and mini-Cas13.

Supplementary Figure 3. Expression and identification of Cas13 proteins.

Supplementary Figure 4. Programmable RNA targeting by RfxCas13d and PspCas13b with their mini variants *in vitro*.

Supplementary Figure 5. The effect of crRNA length and mismatch on RfxCas13d, mini-RfxCas13d and hfRfxCas13d activity.

Supplementary Figure 6. Volcano plot of differential transcript with Cas13d.

Supplementary Figure 7. The overall structure of EsCas13d and mini-EsCas13d.

Supplementary Figure 8. The overall structure of RspCas13d and mini-RspCas13d.

Supplementary Figure 9. Comparison of the HEPN1 and HEPN2 domains of Cas13d.

Supplementary Figure 10. Comparison of the Helical1 and Helical2 domains of Cas13d.

Supplementary Figure 11. Comparison of the HEPN1 and HEPN2 domains of Cas13b.

Supplementary Figure 12. Comparison of the Helical1 and Helical2 domains of Cas13b.

Supplementary Figure 13. Sanger sequencing of mini-Vx and Vx mediated base transversion.

Supplementary Figure 14. Off-target RNA editing effect for Vx and mini-Vx system.

Supplementary Figure 15. The results of mini-RfxCas13d in N2A cells and *in vivo*.

Supplementary Figure 16. Comparison the Δ2 and N3V7 fragments of RfxCas13d.

Supplementary Figure 17. EsCas13d and PbuCas13b colored by conservation of Cas13ds and Cas13bs.

Supplementary Figure 18. Multiple sequence alignment of Cas13d proteins from different species.

Supplementary Figure 19. Multiple sequence alignment of Cas13b proteins from different species.

Supplementary Tables

Supplementary Table 1. Cas13 protein sequences used in this study.

Supplementary Table 2. Primers used in this study.

Supplementary Table 3. CrRNA spacer sequences for RNA knockdown experiments.

Supplementary Table 4. CrRNA sequences used in this study for RNA editing in mammalian cells.

Supplementary Table 5. CrRNA spacer sequences for *Pcsk9* knockdown experiments.

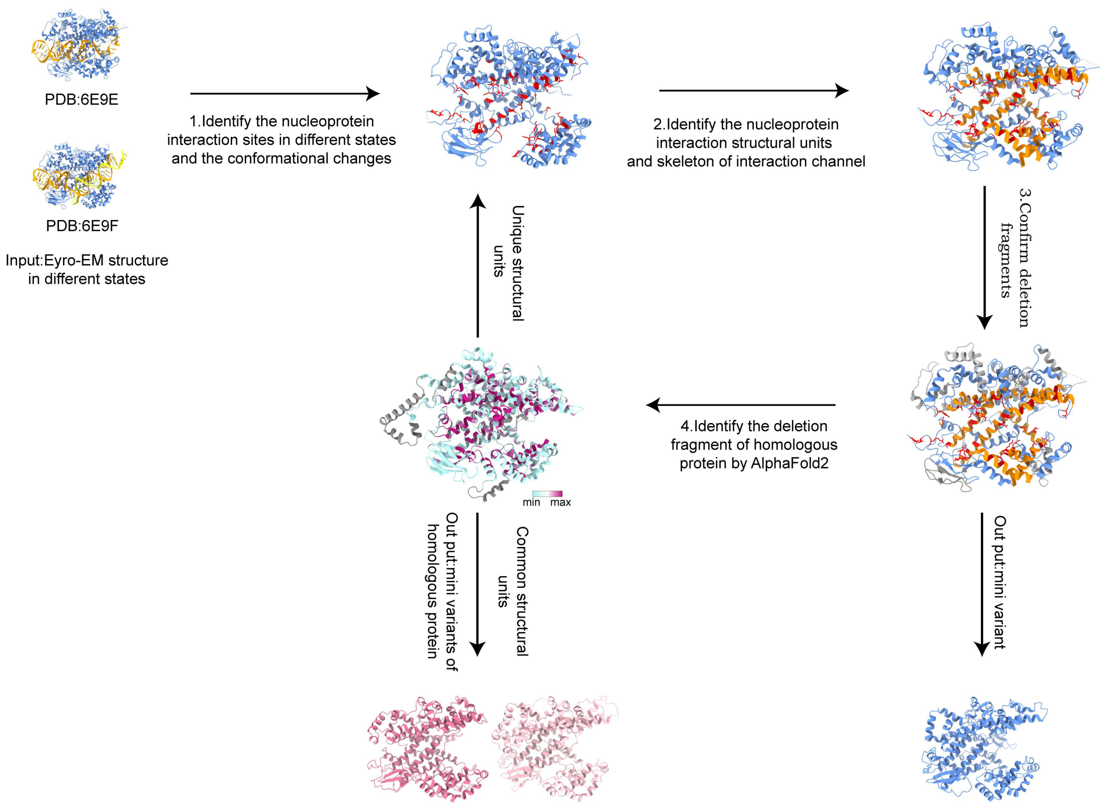

Figure S1. The workflow of IDC strategy.

The interaction sites and the structural units of interaction sites are colored in red and orange.

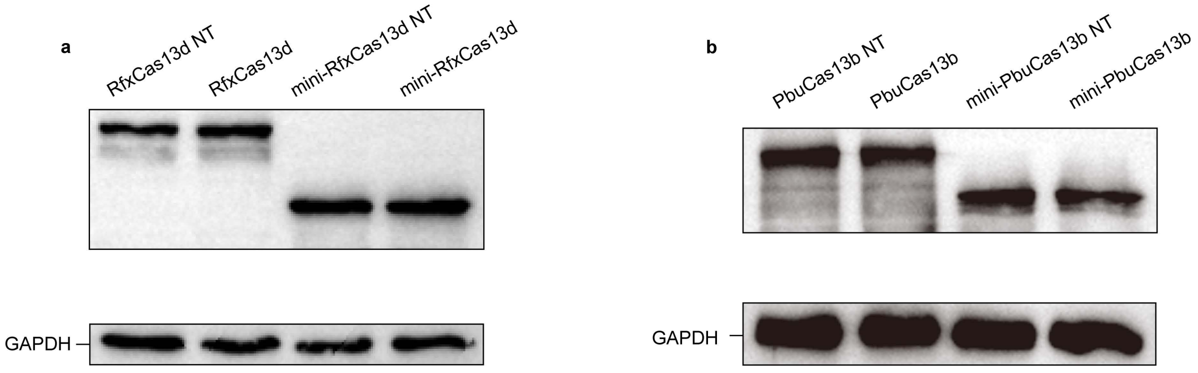

Figure S2. Western blot assay of Cas13 and mini-Cas13.

(a) The protein expression level of RfxCas13d and mini-RfxCas13d. (b) The protein expression level of PbuCas13b and mini-PbuCas13b. (a-b) Source data are provided as a Source Date file.

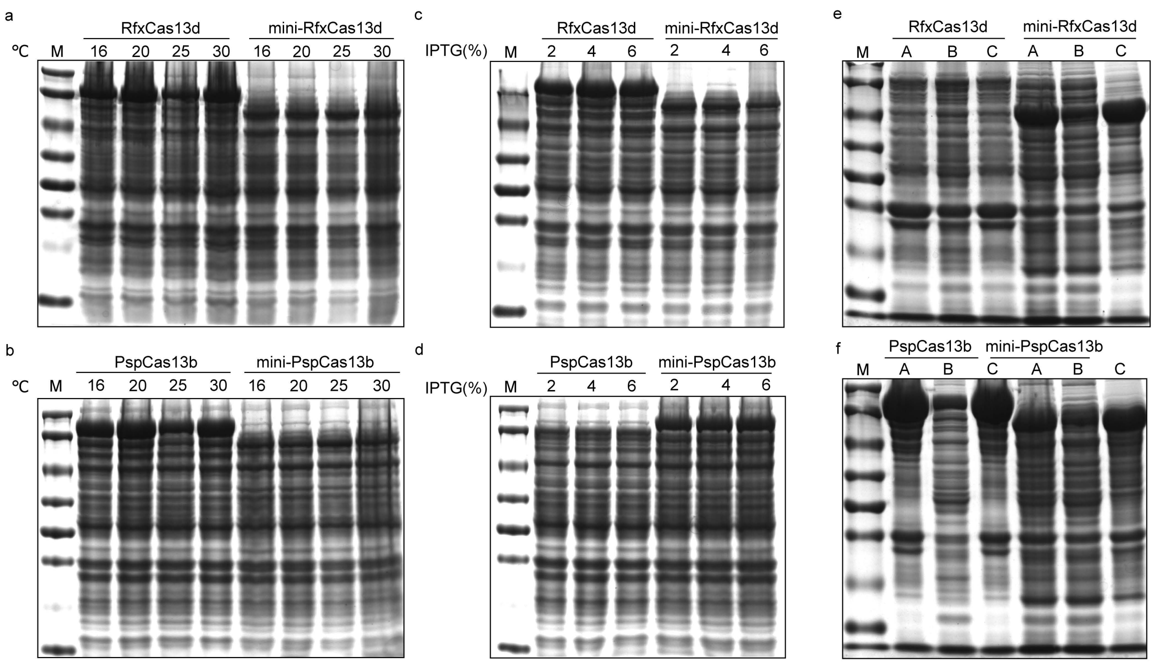

Figure S3. Expression and identification of Cas13 proteins.

(a) Optimal induced temperature range for RfxCas13d, mini-RfxCas13d, PspCas13b, and mini-PspCas13b proteins expression at 2%concentration of IPTG. (b) RfxCas13d, mini-RfxCas13d, PspCas13b, and mini-PspCas13b proteins expression induced at different concentrations of IPTG. (c) Identification of the expression form of RfxCas13d, mini-RfxCas13d, PspCas13b, and mini-PspCas13b proteins. M: Protein Marker; A: Induced recombinant bacteria; B: Induced recombinant bacteria lysed supernatant; C: Induced recombinant bacteria lysed precipitate. (a-c) Source data are provided as a Source Date file.

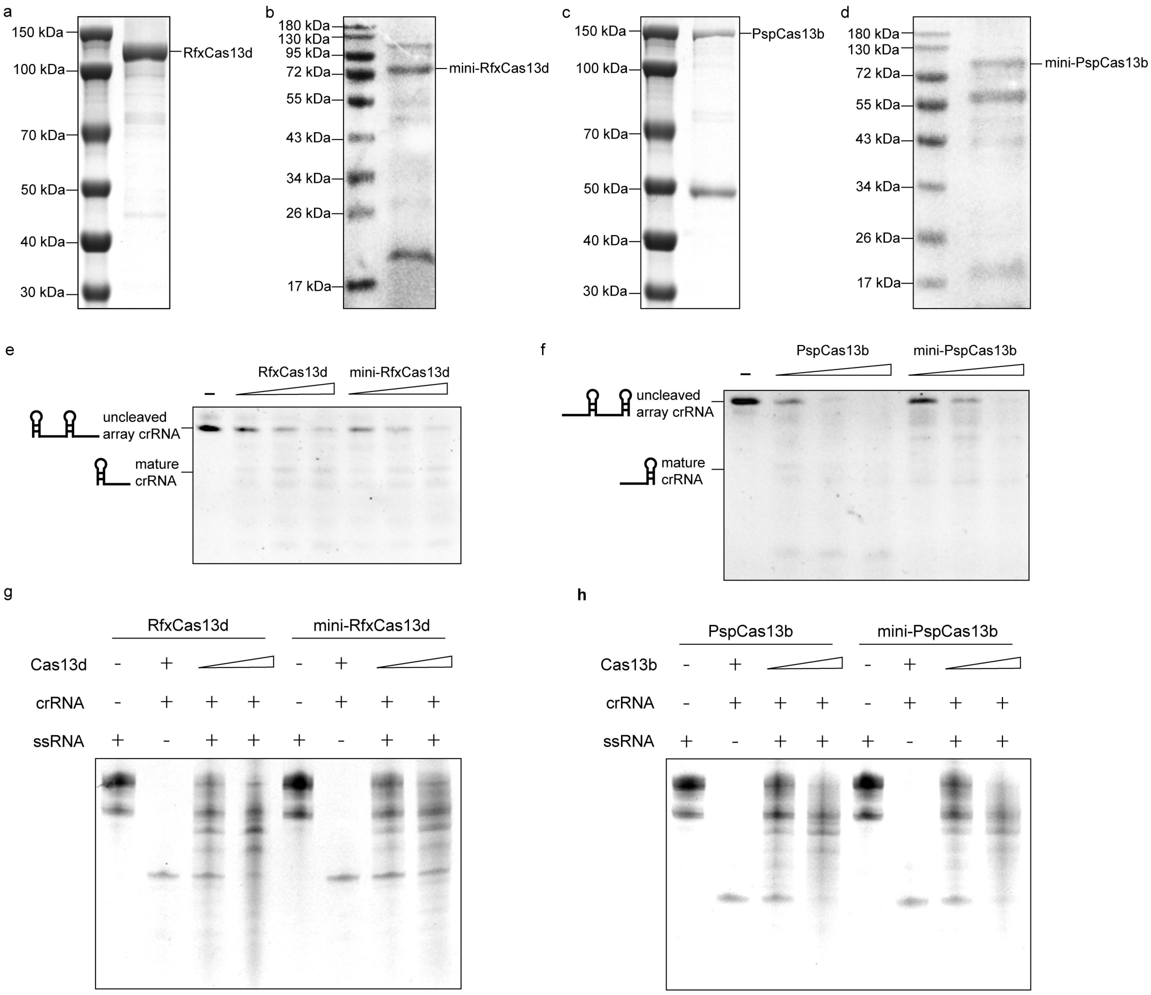

Figure S4. Programmable RNA targeting by RfxCas13d and PspCas13b with their mini variants *in vitro*.

(a-d) Coomassie blue stained polyacrylamide gel of purified recombinant proteins (a)RfxCas13d, (b)mini-RfxCas13d, (c)PspCas13b and (d)mini-PspCas13b respectively, which were used in all biochemical assays.

(e) Representative denaturing gels depicting RfxCas13d and mini-RfxCas13d mediated cleavage of their cognate pre-crRNAs over a dose titration of effector concentration.

(f) Representative denaturing gels depicting PspCas13b and mini-PspCas13b mediated cleavage of their cognate pre-crRNAs over a dose titration of effector concentration.

(g) Representative denaturing gels displaying the targeted RNase activity of RfxCas13d and mini-RfxCas13d effector proteins, with substrate RNA cleavage occurring when the crRNA matches its complementary target ssRNA.

(h) Representative denaturing gels displaying the targeted RNase activity of PspCas13b and mini-PspCas13b effector proteins, with substrate RNA cleavage occurring when the crRNA matches its complementary target ssRNA. (a-h) Source data are provided as a Source Date file.

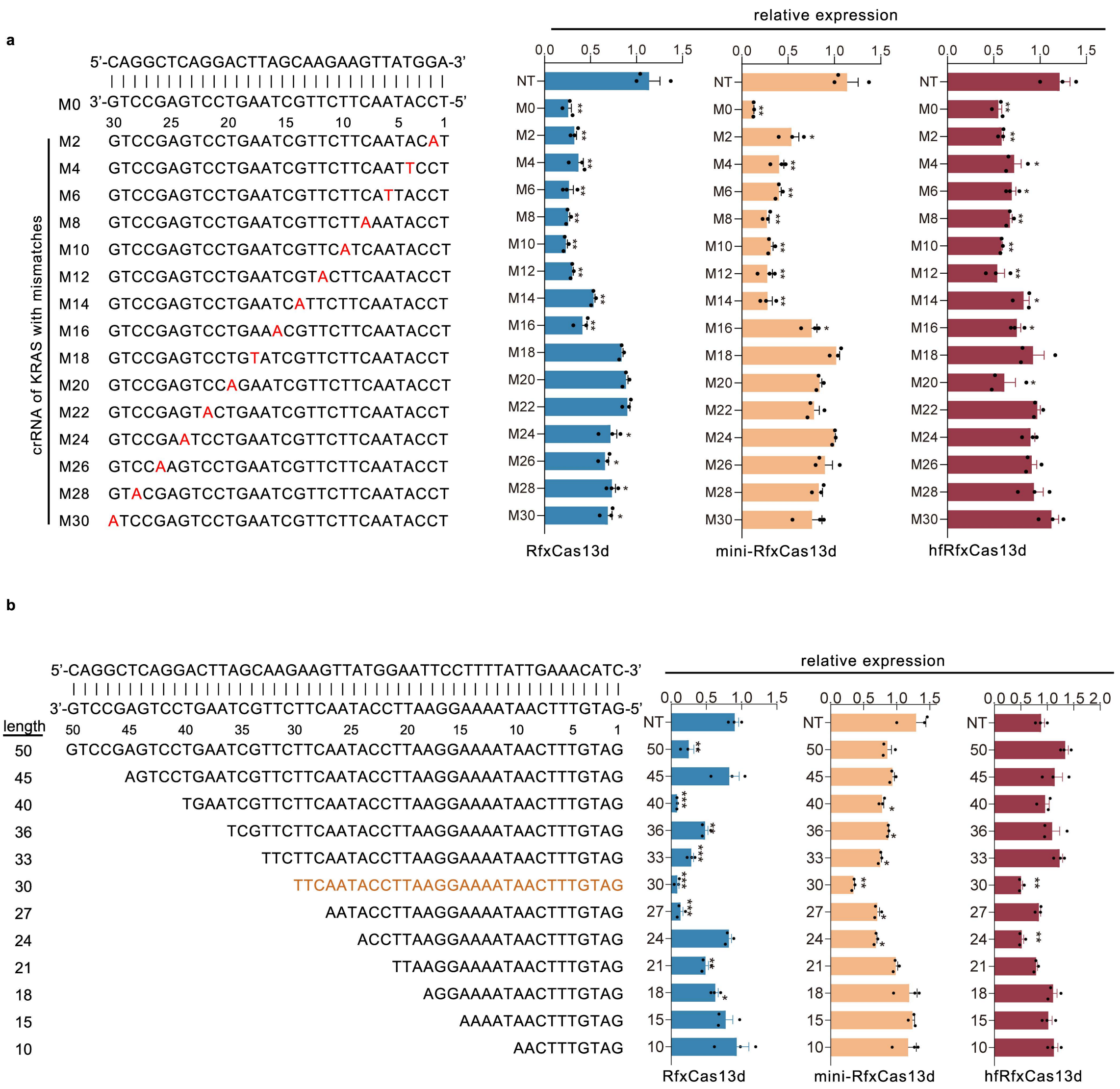

Figure S5. The effect of crRNA length and mismatch on RfxCas13d, mini-RfxCas13d and hfRfxCas13d activity.

(a)Left, *KRAS* crRNAs of different single-nucleotide mismatches; right, relative expression of *KRAS* transcript by co-transfection with RfxCas13d, mini-RfxCas13d, or hfRfxCas13d. (b)Left, *KRAS* crRNAs of different lengths; right, relative expression of *KRAS* transcript by co-transfection of RfxCas13d, mini-RfxCas13d, or hfRfxCas13d. The data are presented as the mean ± SD. Two-tailed unpaired two-sample t-test. *P < 0.05, **P < 0.01, ***P < 0.001, ns, not significant. Source data are provided as a Source Date file.

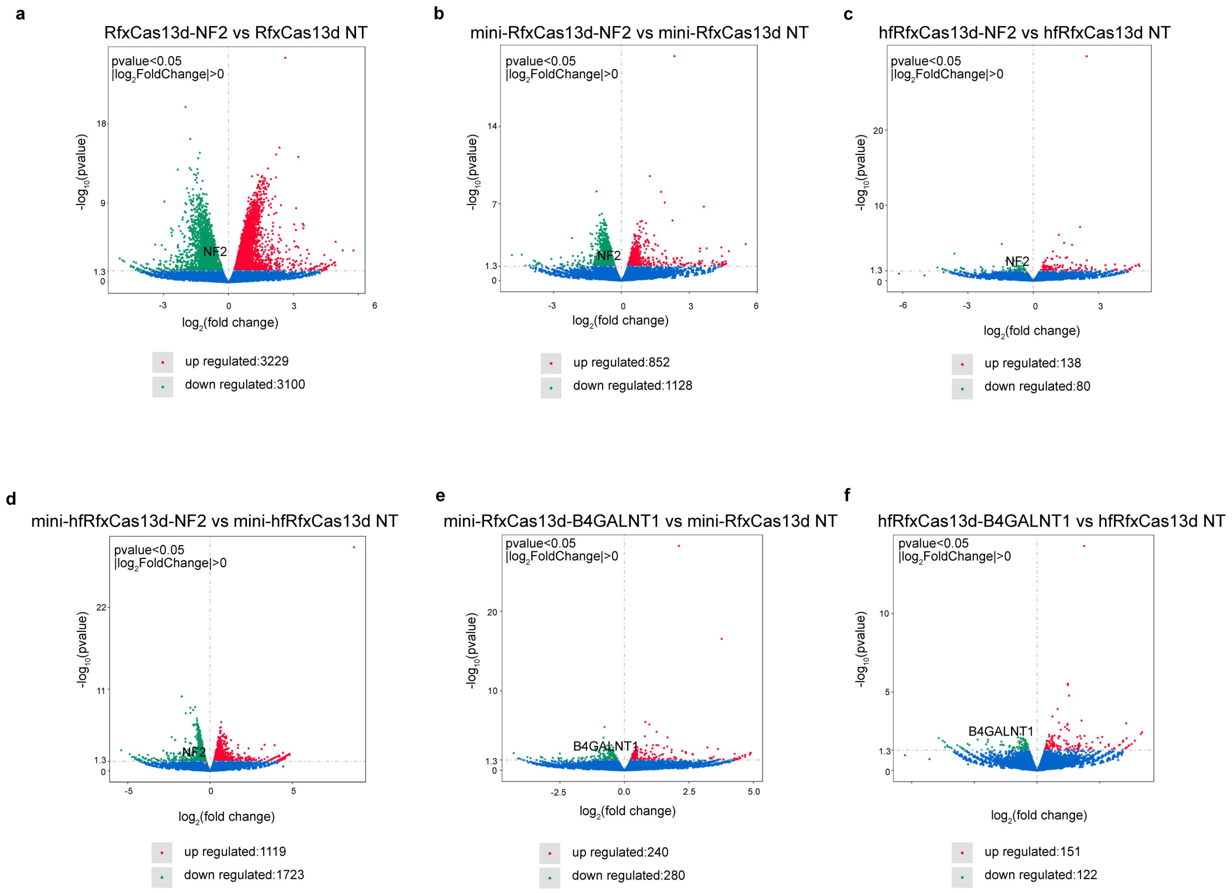

Figure S6. Volcano plot of differential transcript with Cas13d.

(a-d) Volcano plot of differential expression genes between *NF2* targeting and non-targeting (NT) by (a)RfxCas13d, (b) mini-RfxCas13d and (c) hfRfxCas13d (d) mini-hfRfxCas13d (n = 3).

(e-f) Volcano plot of differential expression genes between *B4GALNT1* targeting and non-targeting (NT) by (d) mini-RfxCas13d and (e) hfRfxCas13d (n = 3).

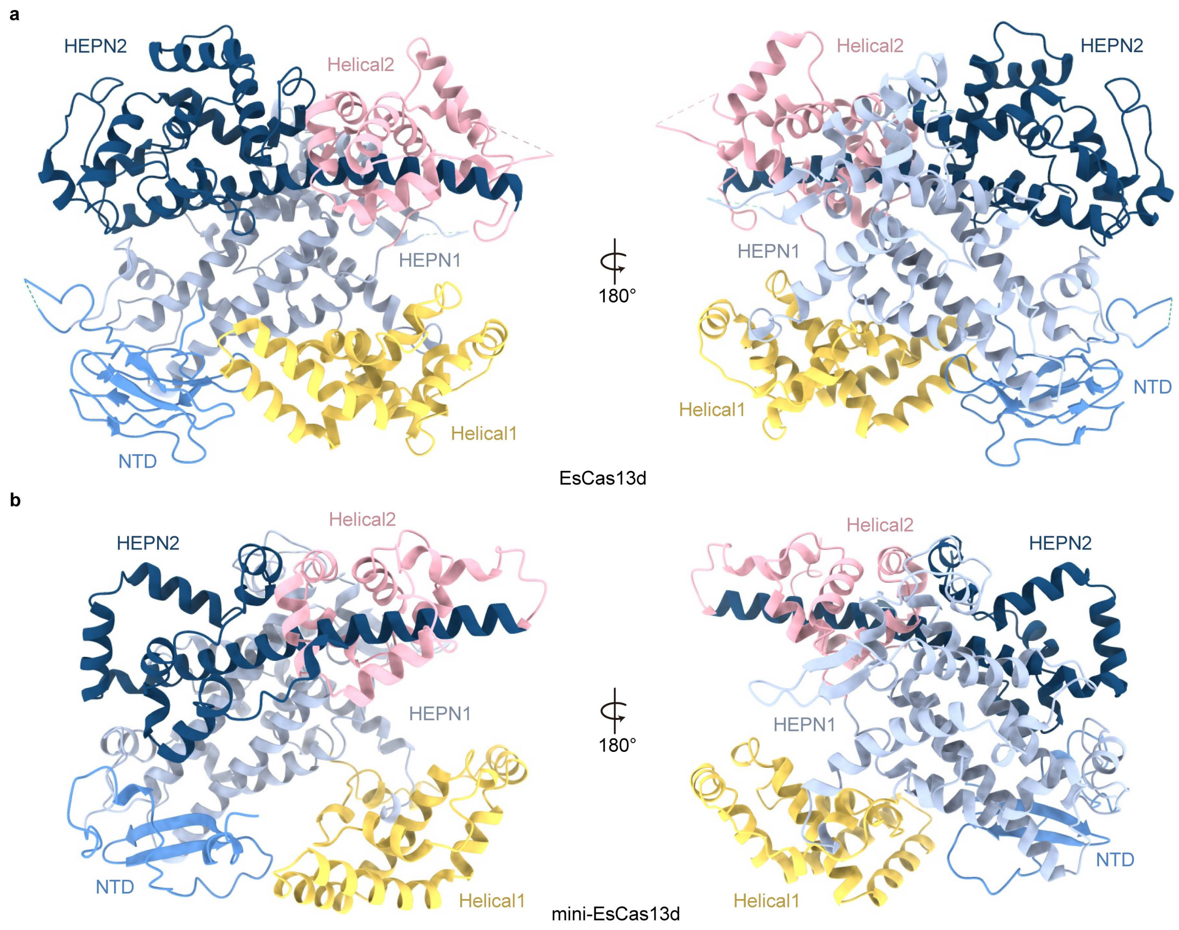

Figure S7. The overall structure of EsCas13d and mini-EsCas13d.

The cryo-electron microscopy structure of (a)EsCas13d (PDB: 6E9E) and predicted overall structure of (b)mini-EsCas13d as indicated by AlphaFold2 are shown in two different orientations and color coded as indicated in Figure 3c.

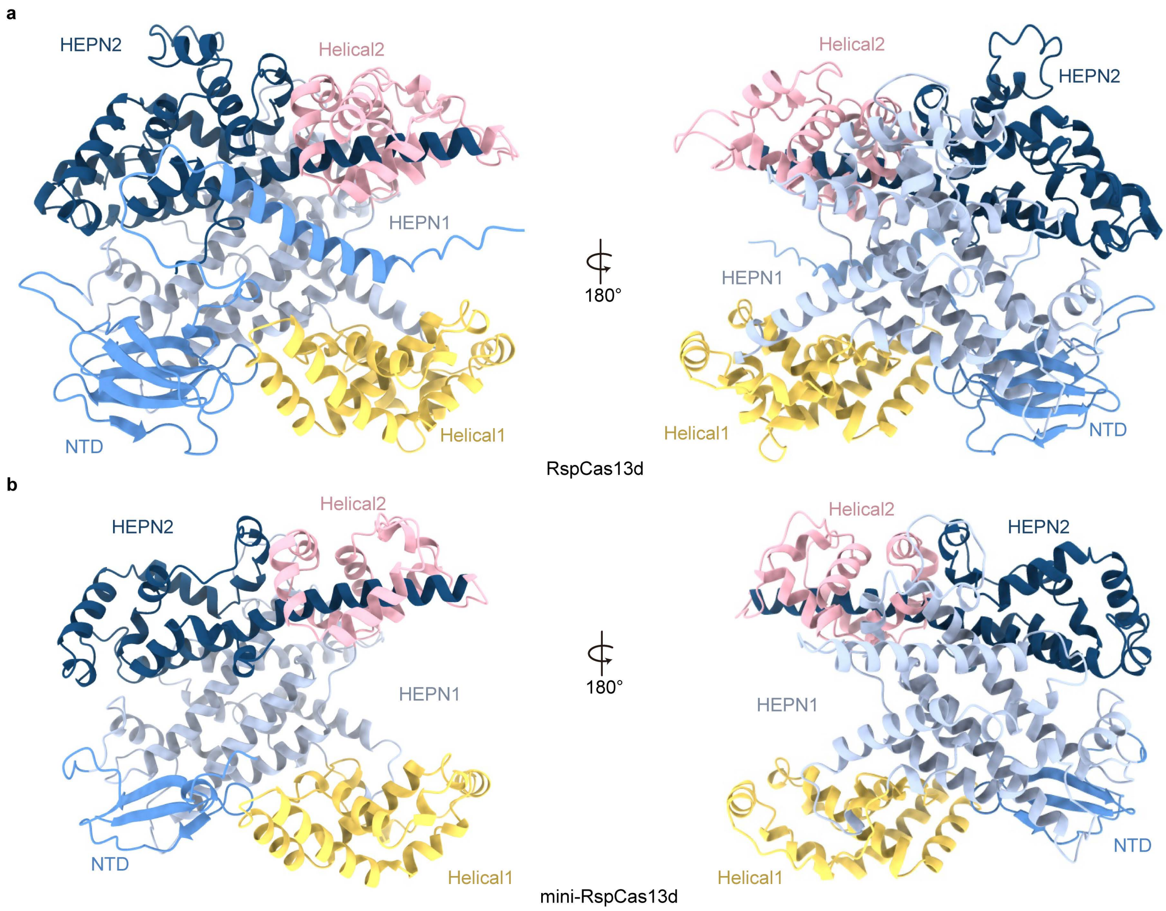

Figure S8. The overall structure of RspCas13d and mini-RspCas13d.

The predicted overall structure of (a)RspCas13d and (b)mini-RspCas13d by AlphaFold2, which is shown in two different orientations and color coded as indicated in Figure 3d.

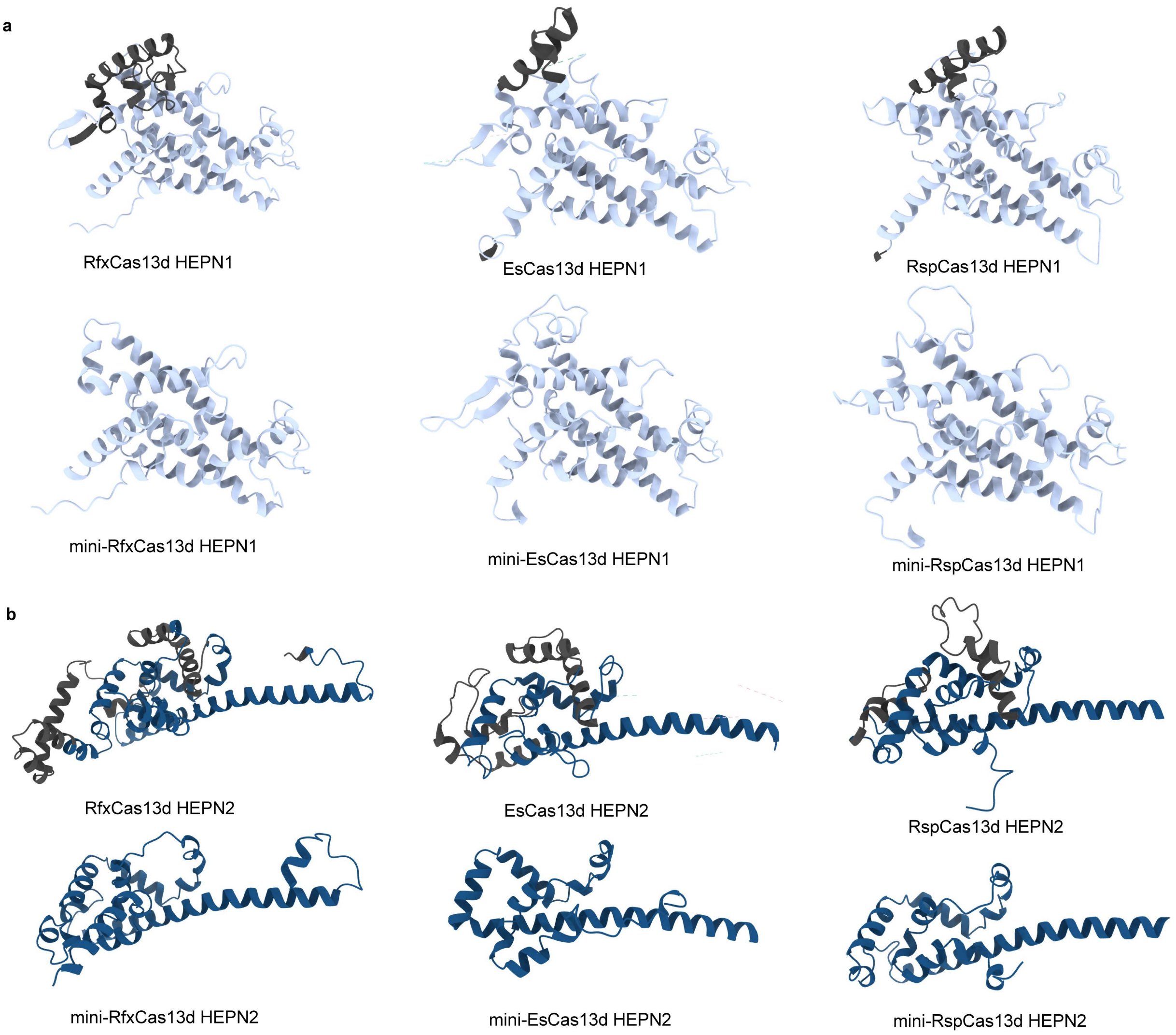

Figure S9. Comparison of the HEPN1 and HEPN2 domains of Cas13d.

(a) The HEPN1 domains of RfxCas13d, mini-RfxCas13d, EsCas13d (6E9E), mini-EsCas13d, RspCas13d and mini-RspCas13d. (b) The HEPN2 domains of RfxCas13d, mini-RfxCas13d, EsCas13d (6E9E), mini-EsCas13d, RspCas13d and mini-RspCas13d. All deletion regions were colored deep gray.

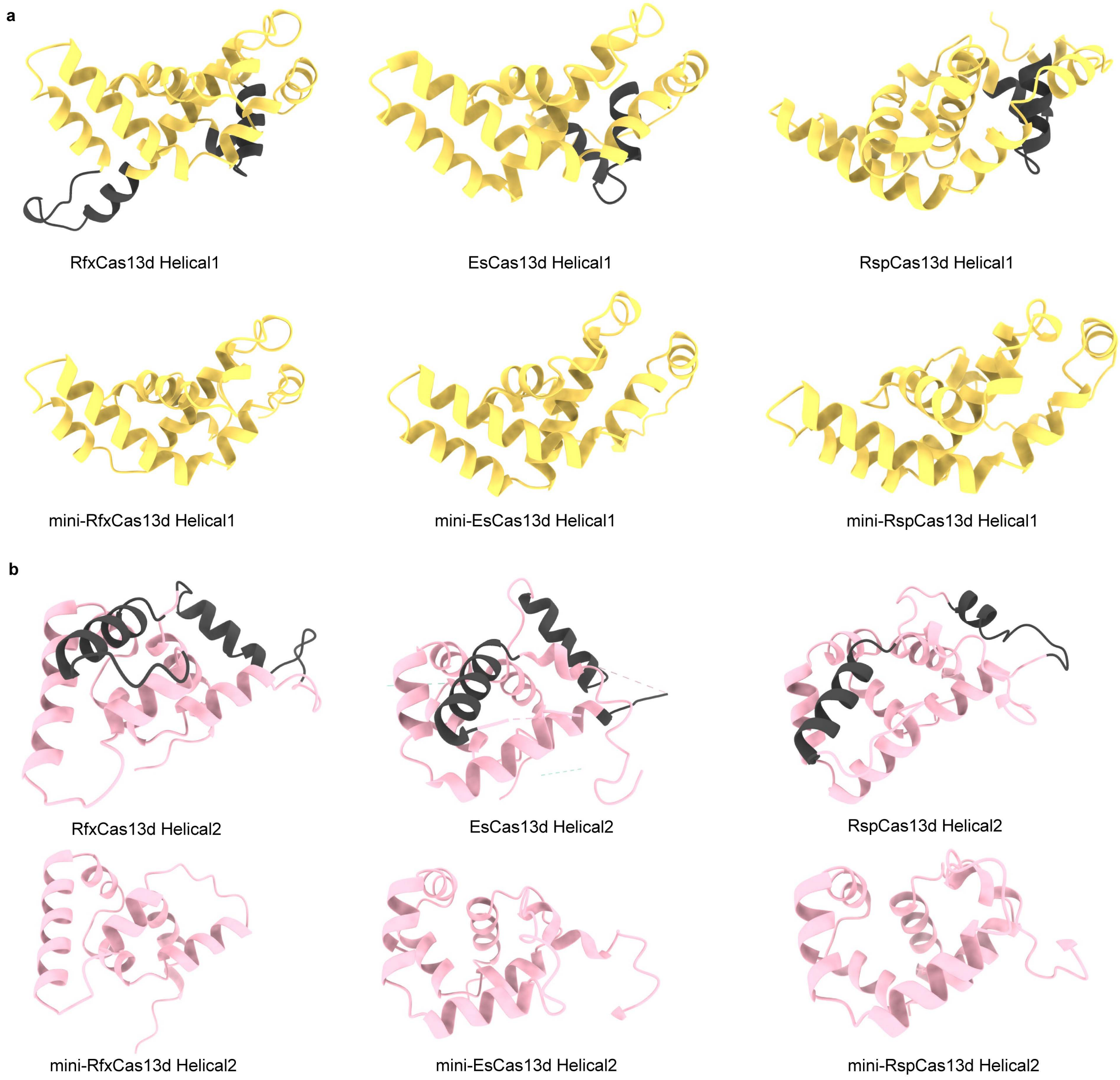

Figure S10. Comparison of the Helical1 and Helical2 domains of Cas13d.

(a) The Helical1 domains of RfxCas13d, mini-RfxCas13d, EsCas13d (6E9E), mini-EsCas13d, RspCas13d and mini-RspCas13d. (b) The Helical2 domains of RfxCas13d, mini-RfxCas13d, EsCas13d (6E9E), mini-EsCas13d, RspCas13d and mini-RspCas13d. All deletion regions were colored deep gray.

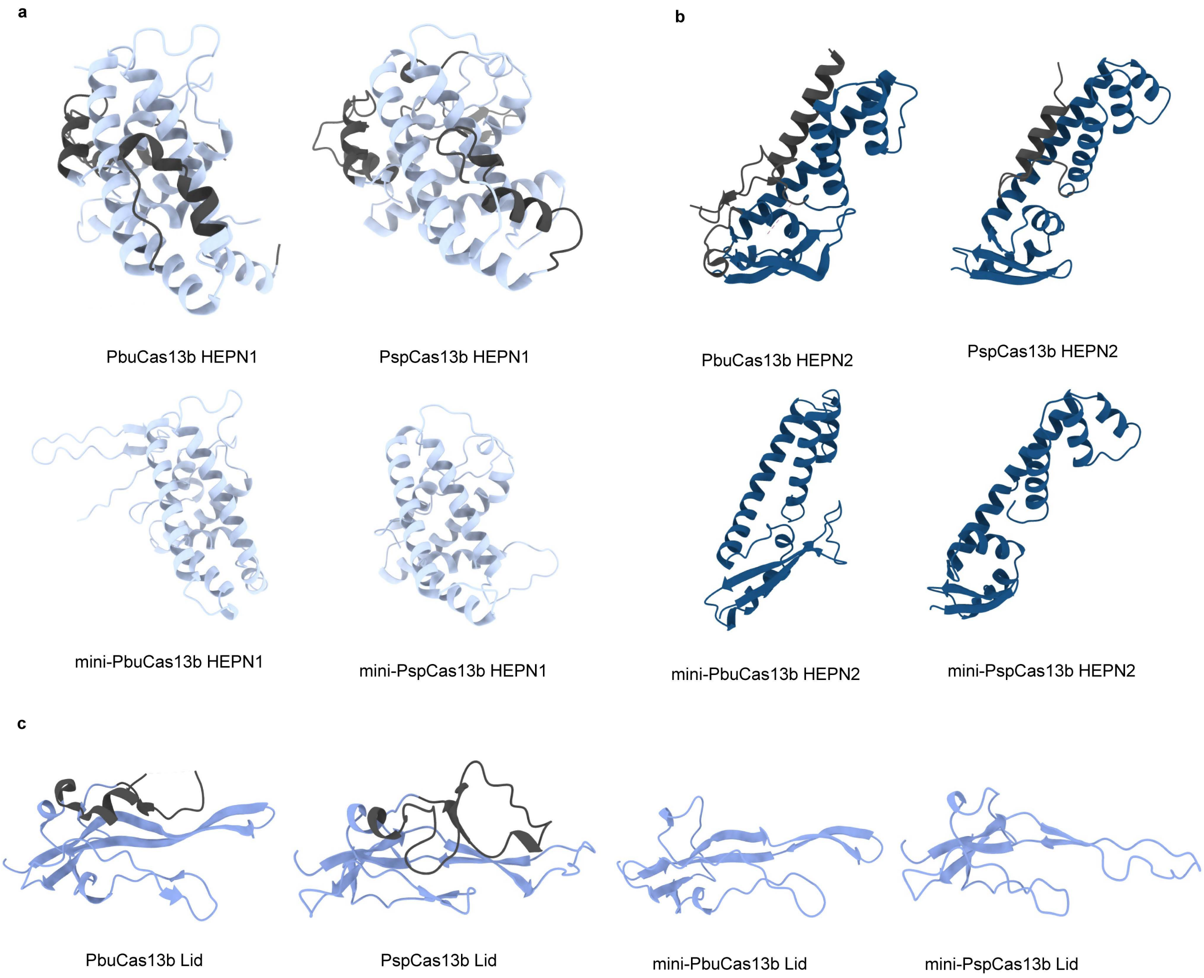

Figure S11. Comparison of the HEPN1 and HEPN2 domains of Cas13b.

(a) The HEPN1 domains of PspCas13b, mini-PspCas13b, PbuCas13b (6DTD), and mini-PbuCas13b. (b) The HEPN2 domains of PspCas13b, mini-PspCas13b, PbuCas13b (6DTD), and mini-PbuCas13b. (c) The Lid domains of PspCas13b, mini-PspCas13b, PbuCas13b (6DTD), and mini-PbuCas13b. All deletion regions were colored deep gray.

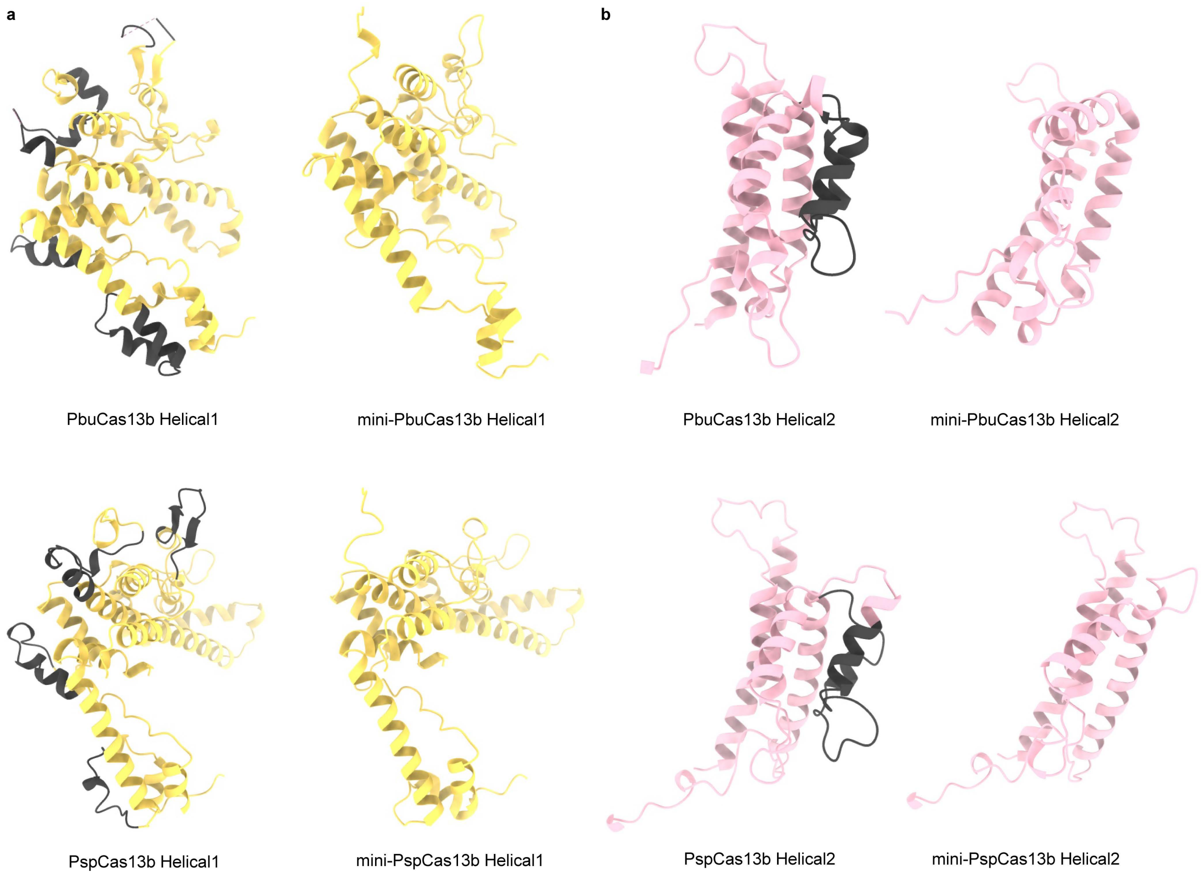

Figure S12. Comparison of the Helical1 and Helical2 domains of Cas13b.

(a) The Helical1 domains of PspCas13b, mini-PspCas13b, PbuCas13b (6DTD), and mini-PbuCas13b. (b) The Helical2 domains of PspCas13b, mini-PspCas13b, PbuCas13b (6DTD), and mini-PbuCas13b. All deletion regions were colored deep gray.

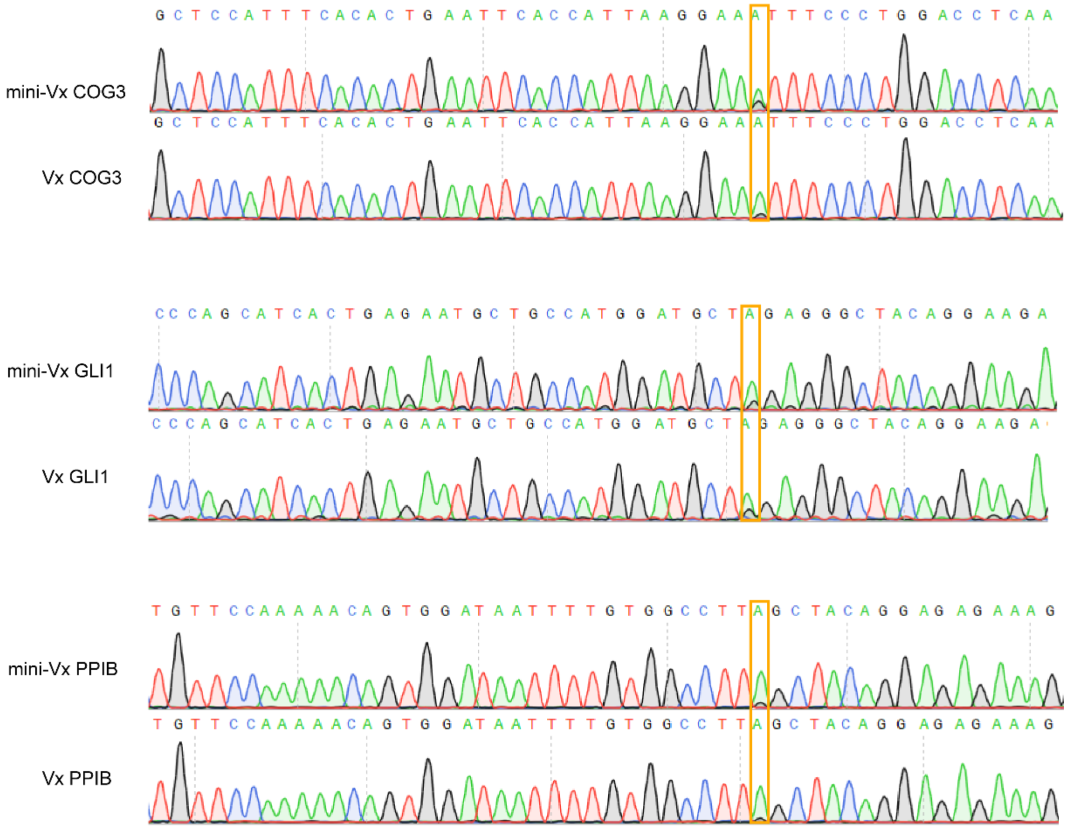

Figure S13. Sanger sequencing of mini-Vx and Vx mediated base transversion. The targeted bases are encompassed by an orange quadrangle. Vx: REPAIRx, mini-Vx: mini-REPAIRx.

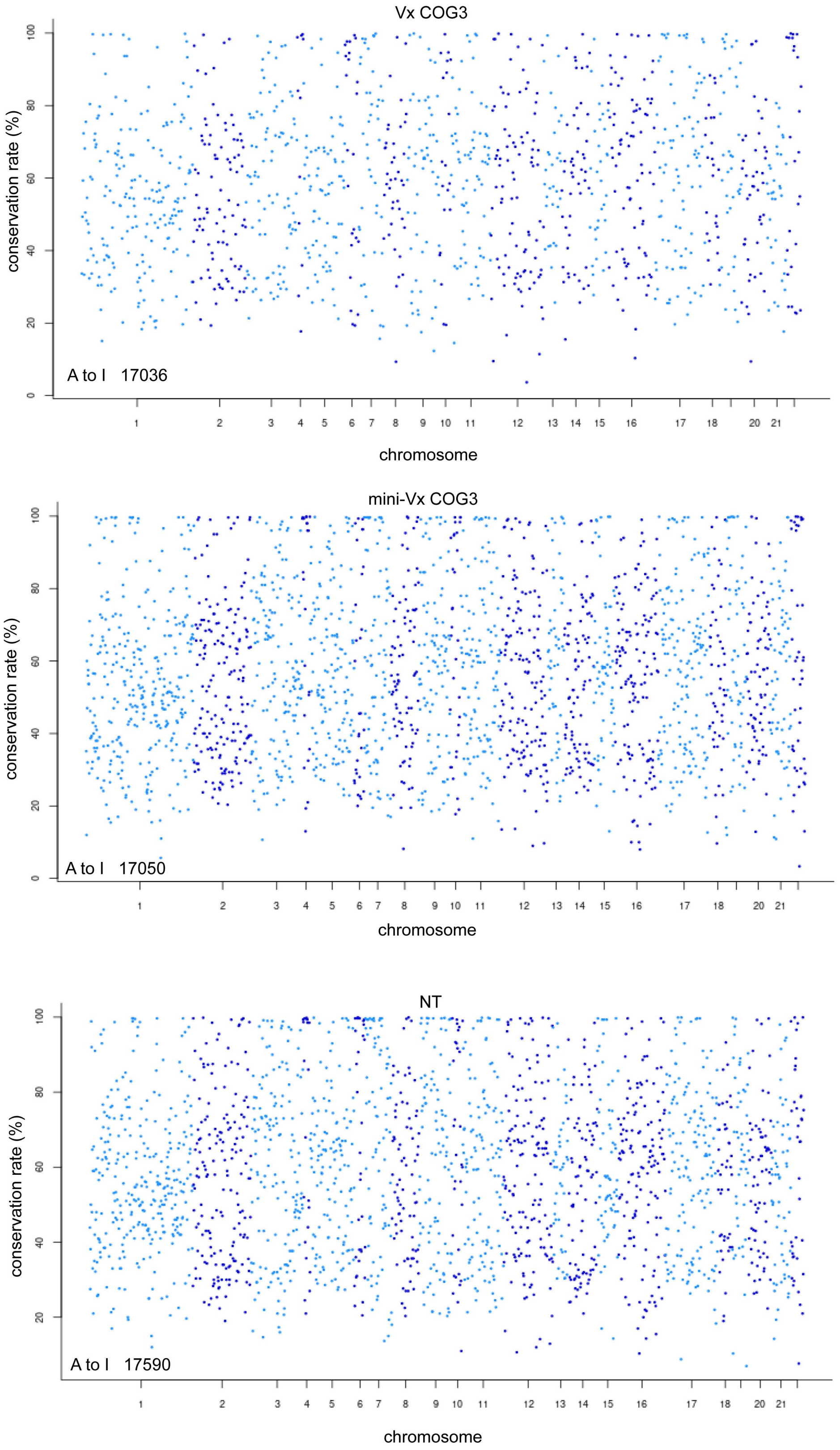

Figure S14. Off-target RNA editing effect for Vx and mini-Vx system. Manhattan plots of transcriptome-wide off-target RNA editing analysis for Vx and mini-Vx with *COG3* targeting guide and non-targeting (NT) guide in HEK293T cells. Vx: REPAIRx, mini-Vx: mini-REPAIRx.

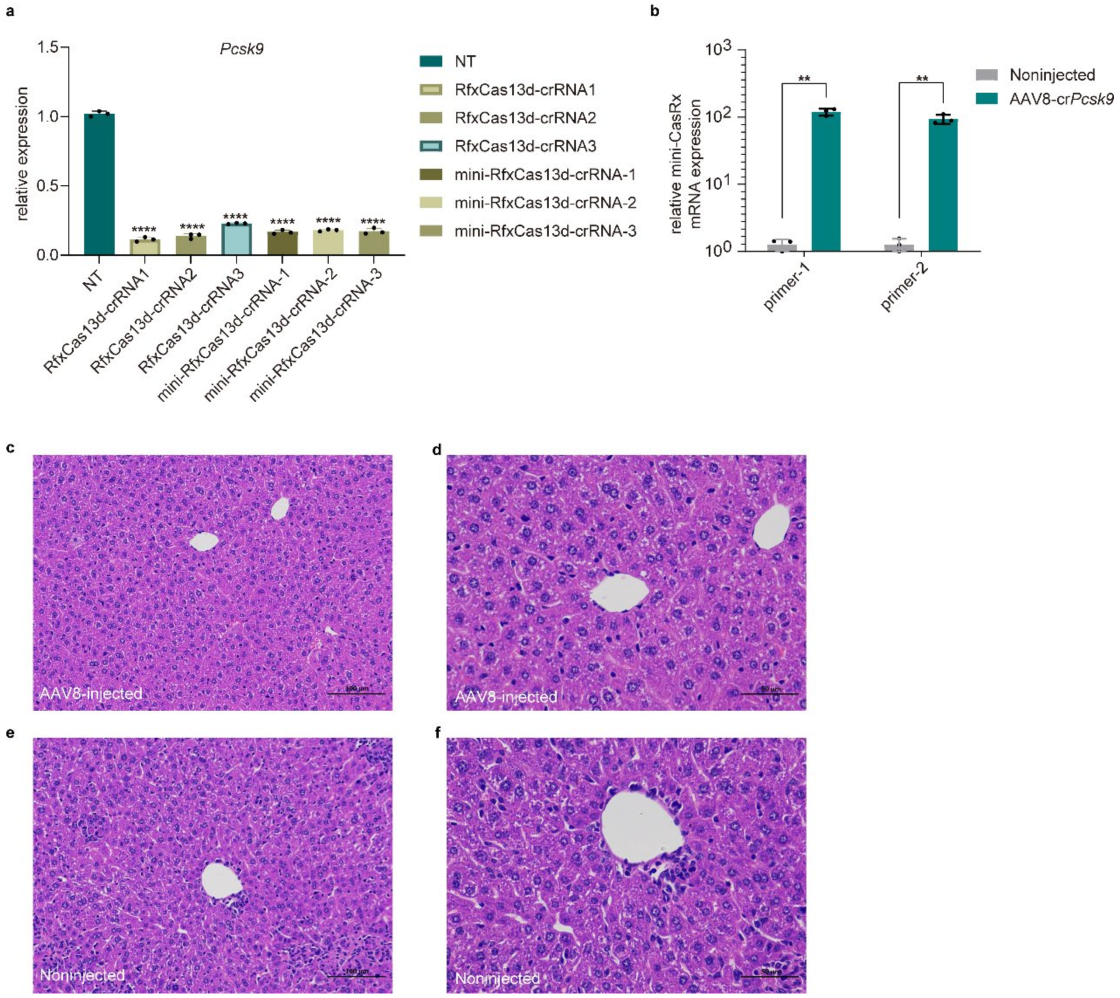

Figure S15. The results of mini-RfxCas13d in N2A cells and *in vivo*.

(a) Quantification of *Pcsk9* mRNA levels in N2a cells. Source data are provided as a Source Date file. (b) Quantification of the mini-RfxCas13d transcript in the liver. NT, non-target crRNA. The data are presented as the mean ± SD. Two-tailed unpaired two-sample t-test. *P < 0.05, **P < 0.01, ***P < 0.001, **** P < 0.0001. (n = 3 biological replicates, each with an average of 3 technical replicates). CasRx: RfxCas13d. Source data are provided as a Source Date file. (c) Morphology of liver slices 21 days after AAV8 injection in mice. The scale bar represents 100 μm in length. (d) A close-up view of (c). The scale bar represents 50 μm in length. (e) Morphology of liver slices from noninjected mice at 21 days. The scale bar represents 100 μm in length. (f) A close-up view of (e). The scale bar represents 50 μm in length.

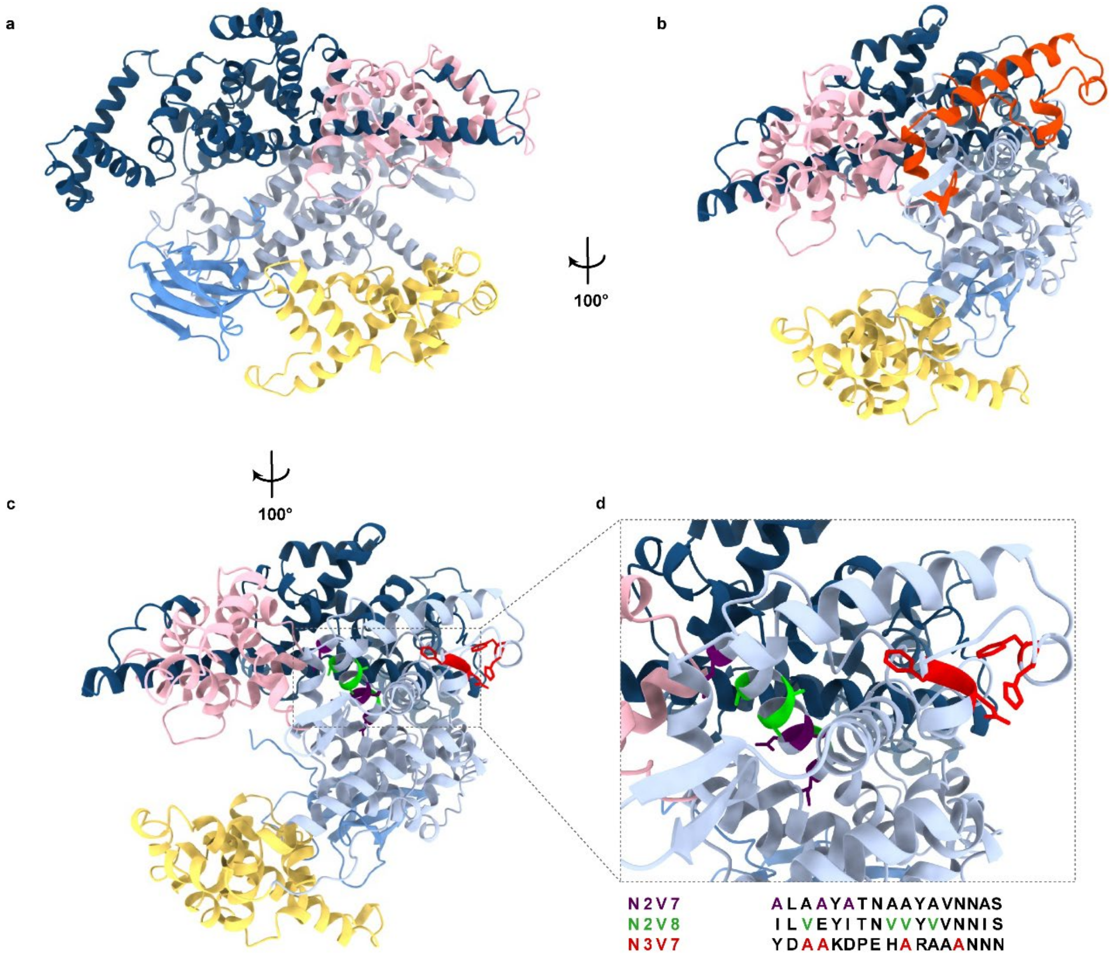

Figure S16. Comparison the Δ2 and N3V7 fragments of RfxCas13d. (a) The predicted structure of RfxCas13d by AlphaFold2 is shown. (b)Positions of Δ2 (orange) in RfxCas13d. (c) Positions of N2V7(purple), N2V8(green), and N3V7 (red) in RfxCas13d. (d) A close-up view of the N2V7(purple), N2V8(green), and N3V7 (red) sequence (red) of RfxCas13d.

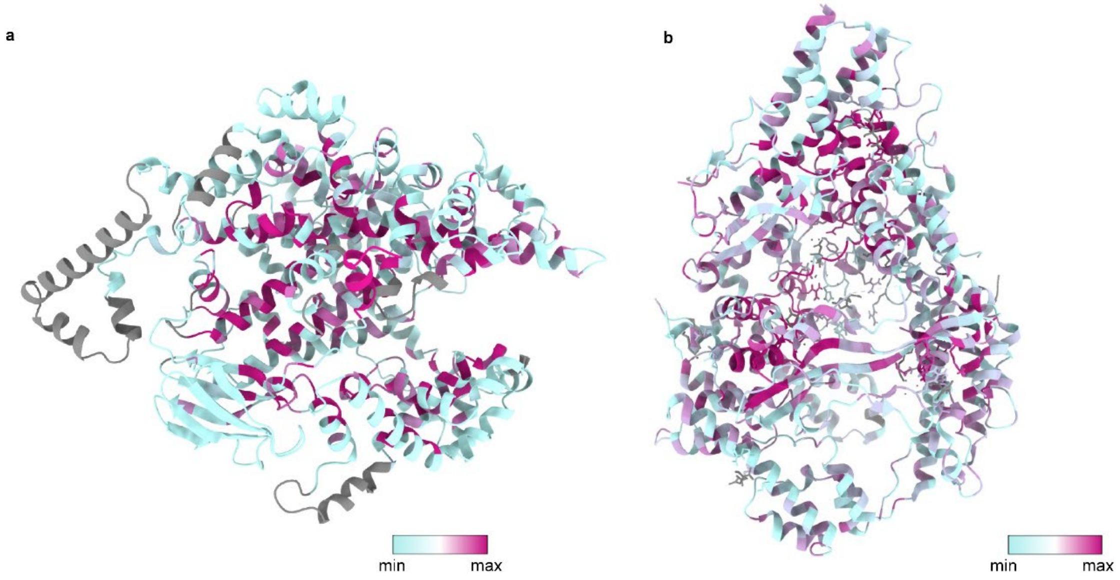

Figure S17. EsCas13d and PbuCas13b colored by conservation of Cas13ds and Cas13bs.

(a) Schematic representation of EsCas13d colored based on conserved sequence percentage. No-value fragments are gray. Regions with a high degree of conservation are concentrated in the RNA-binding cleft and the HEPN active site. Related to Figure S18.

(b) Schematic representation of PbuCas13b colored based on conserved sequence percentage. No-value fragments are gray. Regions with a high degree of conservation are concentrated in the RNA-binding cleft and the HEPN active site. Related to Figure S19.

```
RSP      1  MAKKNKMKPRELREAQKKARQLKAAEINN....NAAPAIAAMPAAEVIAPVAEKKK....
UR       1  MAKKNKMKPRELREAQKKARQLKAAEINN....NAAPAIAAMPAAEVIAPAAEKKK....
RFF      1  ..MKKKMSLREKREAEKQAKKAAYSAASKNTDSKPAEKKAETPKPAEIISDNSRNK....
RA       1  ..MKKKMSLREKREAEKQAKKAAYSAASKNTDSKPAEKKAETPKPAEIISDNSRNK....
P1E0     1  .............................................MEREVKKPPKK....
ES       1  ..MGKKIHARDLREQRKTDRTEKFADQNKKREAERAVPKKDAAVSVKSVSSVSSKKDNVT
RFX      1  ..................................................MIEKKK....
ADM      1  ..................................................MNNKRK....

RSP     53  .SSVKAAGMKSILVS...KNKMYITSFGKGNSAVLEYEVDNNDYNQTQLSSKGSSNIELR
UR      53  .SSVKAAGMKSILVS...ENKMYITSFGKGNSAVLEYEVDNNDYNQTQLSSKDNSNIQLG
RFF     55  .TAVKAAGLKSTIIS...GDKLYMTSFGKGNAAVIEQKIDINDYSFSAMKDTPS..LEVD
RA      55  .TAVKAAGLKSTIIS...GDKLYMTSFGKGNAAVIEQKIDINDYSFSAMKDTPS..LEVD
P1E0    12  .SLAKAAGLKSTFVISPQEKELAMTAFGRGNDALLQKRIVDG.....VVRDVAGEKQQFQ
ES      59  KSMAKAAGVKSVFAVG...NTVYMTSFGRGNDAVLEQKIVDTSHEPLNIDDPAY...QLN
RFX      7  .SFAKGMGVKSTLVSG...SKVYMTTFAEGSDARLEKIVEGDSIRSVNEGEAFS..AEMA
ADM      7  .TKAKAAGLKSVFFDQ...KQAVLTTFAKGNNSQIEKKVVNSEVKDLRQPPAFDLELKEK

RSP    109  GVNEVN............ITFSSKHGFESGVEINTSNPTHRSGESS..PVRG..DMLGLK
UR     109  GVNEVN............ITFSSKHGFESGVEINTSNPTHRSGESS..PVRG..DMLGLK
RFF    109  KAESKE............ISFSSHHPFVKNDKLTTYNPLYGGKDNPEKPVGR..DMLGLK
RA     109  KAESKE............ISFSSHHPFVKNDKLTTYNPLYGGKDNPEKPVGR..DMLGLK
P1E0    66  VQRQDE............SRFRLQNSRLADRTVTADDPLHRAETPRRQPLGAGMDQLRRK
ES     113  VVTMNG............YSVTGHRGETVSAVTDNPLRRFNGRKKDEPEQSVPTDMLCLK
RFX     61  DKNAGY............KIGNAKFSHPKGYAVVANNPLYTG........PVQQDMLGLK
ADM     63  TFYISGKNNINTSRENPLASASLPLSKRQRIRAERIKRAREENRPYHNVKRVGEDDLRAK

RSP    153  SELEKRFFGKTFD..DNIHIQLIYNILDIEKILAVYVTNIVYALNNMLSIKDS.ESYDDF
UR     153  SELEKRFFGKTFD..DNIHIQLIYNILDIEKILAVYVTNIVYALNNMLGVKGS.ESHDDF
RFF    155  DKLEERYFGCTFN..DNLHIQIIYNILDIEKILAVHSANITTALDHMVDEDDEKYLNSDY
RA     155  DKLEERYFGCTFN..DNLHIQIIYNILDIEKILAVHSANITTALDHMVDEDDEKYLNSDY
P1E0   114  AILEQKYFGRTFD..DNIHIQLIYNILDIHKMLAVPANHIVHTLNLLGG.....YGETDF
ES     161  PTLEKKFFGKEFD..DNIHIQLIYNILDIEKILAVYSTNAIYALNNMSADEN..IENSDF
RFX    101  ETLEKRYFGESADGNDNICIQVIHNILDIEKILAEYITNAAYAVNNISGLDKDIIGFGKF
ADM    123  ADLEKHYFGKEYS..DNLKIQIIYNILDINKIISPYINDIVYSMNNLARNDEYIDGKIDV

RSP    210  MGYLSARNTYEVFTHPD.KSNLSDKAKGNIKKSFSTFNDLLKTKRLG........YFGLE
UR     210  IGYLSTNNIYDVFIDPD.NSSLSDDKKANVRKSLSKFNALLKTKRLG........YFGLE
RFF    213  IGYMNTINTYDVFMDPSKNSSLSPKDRKNIDNSRAKFEKLLSTKRLG........YFGFD
RA     213  IGYMNTINTYDVFMDPSKNSSLSPKDRKNIDNSRAKFEKLLSTKRLG........YFGFD
P1E0   167  VGMLPAGLPYDKLRVVKKKNGDTVDIKADIA.AYAKRPQLAYLGAAF........YDVTP
ES     217  FMKRTTDETFDDFEKKKESTNSREKADFDAFEKFIGNYRLAYFADAF........YVNKK
RFX    161  S.TVYTYDEFKDPEHHRAAFNNNDKLINAIKAQYDEFDNFLDNPRLG........YFG.Q
ADM    181  IGSLSSTTDYSSFMSPNKDLEKEKKFSFHRENYKKFVEASKPYMRYYGKVFIRDVKKSKL

RSP    261  EPK.TKDTRVSQAYKKRVYHMLAIVGQIRQSVFHDKS...SKLDEDLYSFIDIIDSEYRE
UR     261  EPK.TKDNRVSQAYKKRVYHMLAIVGQIRQCVFHDKS...GAKRFDLYSFINNIDPEYRD
RFF    265  YDANGKDKKKNEEIKKRLYHLTAFAGQLRQWSFHSAG...NYPRTWLY.KLDSLDKEYLD
RA     265  YDANGKDKKKNEEIKKRLYHLTAFAGQLRQWSFHSAG...NYPRTWLY.KLDSLDKEYLD
P1E0   218  GKSKRDAARGRVKREQDVYAILSLMSLLRQFCAHDSVRIWGQNTTAALYHLQALPQDMKD
ES     269  NPKGKAKNVLRE..DKELYSVLTLIGKLRHWCVHSEE....GRAEFWLYKLDELKDDFKN
RFX    211  AFFSKEGRNYIINYGNECYDILALLSGLRHWVVHNNEEESRISRTWLYNLDKNLDNEYIS
ADM    241  STGKGEKIEVMYRSDEEIFTIFQILSYVRQSIMHNDIG.....NKSSILAIEKYPARFVG

RSP    317  TLDYLVDERFDSINKGFIQGN.KVNISLLIDMMKGYEADD....IIRLYYDFIVLKSQKN
UR     317  TLDYLVEERLKSINKDFIEDN.KVNISLLIDMMKGYEADD....IIRLYYDFIVLKSQKN
RFF    321  TLDHYFDKRFNDINDDFVTKN.ATNLYILKEVFPEANFKD....IADLYYDFIVIKSHKN
RA     321  TLDHYFDKRFNDINDDFVTKN.ATNLYILKEVFPEANFKD....IADLYYDFIVIKSHKN
P1E0   278  LLDDGWRRALGGVNDHFLDTN.KVNLLTLFEYYGAETKQARV.ALTQDFYRFVVLKEQKN
ES     323  VLDVVVYNRPVEEINNRFIENN.KVNIQILGSVYKNTDIAE....LVRSYYEFLITKKYKN
RFX    271  TLNYLYLDRITNELTNSFSKNS.AANVNYIAETLGINPAEF....AEQYFRFSIMKEQKN
ADM    296  FLSDLLKTKTNDVNRMFIDNNSQTNFWVLFSIFGLQDHTSGADKICRNFYDFVIKADSKN
```

```
RSP   372  LGFSIKKLREKMLDEYGFR.FKDKQ..YDSVRSKMYKLMDFLLFCNYYRN..........
UR    372  LGFSIKKLREKMLDEYGFR.FKDKQ..YDSVRSKMYKLMDFLLFCNYYRN..........
RFF   376  MGFSIKKLREKMLECDGADRIKEQD..MDSVRSKLYKLIDFCIFKYYHEF..........
RA    376  MGFSIKKLREKMLECDGADRIKEQD..MDSVRSKLYKLIDFCIFKYYHEF..........
P1E0  336  MGFSLRRLREELLKLPDAAYLTGQE..YDSVRQKLYMLLDFLLCRLYAQE..........
ES    378  MGFSIKKLRESMLEG...KGYADKE..YDSVRNKLYQMTDFILYTGYINE..........
RFX   325  LGFNITKLREVMLDRKDMSEIRKNHKVFDSIRTKVYTMMDFVIYRYYIEEDAKVAAANKS
ADM   356  LGFSLKKIRELMLDLPNANMLRDHQ..FDTVRSKFYTLLDFIIYQHYLEE..........

RSP   419  ......DVVAGEALVRKLRFSMTDDEKEGIYADEASKLWGKFRNDFENIADHMNGDVIKE
UR    419  ......DIAAGESLVRKLRFSMTDDEKEGIYADEAAKLWGKFRNDFENIADHMNGDVIKE
RFF   424  ......PELS.EKNVDILRAAVSDTKKDNLYSDEAARLWSIFKEKFLGFCDKI...VVWV
RA    424  ......PELS.EKNVDILRAAVSDTKKDNLYSDEAARLWSIFKEKFLGFCDKI...VVWV
P1E0  384  ......RADRCEELVSALRCALSDEEKDTVYQAEAAALWQALGDTLRRKLLPLLKGKKLQ
ES    423  ......DSDRADDLVNTLRSSLKEDDKTTVYCKEADYLWKKYRESIREVADALDGDNIKK
RFX   385  LPDNEKSLSEKDIFVINLRGSFNDDQKDALYYDEANRIWRKLENIMHNIKEFRGNKTREY
ADM   404  .......KSRIDNMVEKLRMTLKEEEKEVLYAAEAKIVWNAIGAKVINKLVPMMNGDALK

RSP   473  LGKADMDFDEKILDSEKK..........NASDLLYFSKMIYMLTYFLDGKEINDLLTTLI
UR    473  LGKADMDFDEKILDSEKK..........NASDLLYFSKMIYMLTYFLDGKEINDLLTTLI
RFF   474  TGEHEKDITSVIDKDAYR..........NRSNVSYFSKLMYAMCFFLDGKEINDLLTTLI
RA    474  TGEHEKDITSVIDKDAYR..........NRSNVSYFSKLMYAMCFFLDGKEINDLLTTLI
P1E0  438  DKDKKKSDELGLSRDVLDGVLFRPAQQGSRANADYFCRLMHLSTWFMDGKEINTLLTTLI
ES    477  LSKSNIEIQEDKLRKCFIS.........YADSVSEFTKLIYLLTRFLSGKEINDLVTTLI
RFX   445  KKKDAPRLPRILP...............AGRDVSAFSKLMYALTMFLDGKEINDLLTTLI
ADM   457  EIKRKNRDRKLPQSVIATVQVN........SDANVFSGLIYFLTLFLDGKEINEMVSNLI

RSP   523  SKFDNIKEFLKIMKSSAVDVEC............................ELTAGYKLFN
UR    523  SKFDNIKEFLKIMKSSAVDVEC............................ELTAGYKLFN
RFF   524  NKFDNIANQIKTAK..ELGINT............................AFVKNYDFFN
RA    524  NKFDNIANQIKTAK..ELGINT............................AFVKNYDFFN
P1E0  498  SKLENIDSLRSVLES..MGLAY............................SFVPAYAMFD
ES    528  NKFDNIRSFLEIMD..ELGLDR............................TFTAEYSFFE
RFX   490  NKFDNIQSFLKVMP..LIGVNA............................KFVEEYAFFK
ADM   509  TKFENIDSLLHVDREIYKSDEKDLDLEIEKLALFFKGVVRPNAKTDTGAGEISKSFSIFQ

RSP   555  DSQRITNELFIVKNIASMRKPASSAKLTMFRDALTILGIDDN..ITDDRISEILKLKEKG
UR    555  DSQRITNELFIVKNIASMRKPAASAKLTMFRDALTILGIDDK..ITDDRISGILKLKEKG
RFF   554  HSEKYVDELNIVKNIARMKKPSSNAKKAMYHDALTILGIPED..MDEKALDEELDLILEK
RA    554  HSEKYVDELNIVKNIARMKKPSSNAKKAMYHDALTILGIPED..MDEKALDEELDLILEK
P1E0  528  HSRYIAGQLRVVNNIARMRKPAIGAKREMYRAAVVLLGVDSP..EAAAAITDDLLQIDPE
ES    558  GSTKYLAELVELNSFVKSCSFDINAKRTMYRDALDILGIESD..KTEEDIEKMIDNILQI
RFX   520  DSAKIADELRLIKSFARMGEPIADARRAMYIDAIRILGTNLS..YDELKALADTFSLDEN
ADM   569  SAERIIEELKFIKNVTRMDN.EIFPSEGVFLDAANVLGVRGDDFDFSNEFVGDDLHSDAN

RSP   613  K..........GIHGLRNFITNNVIESSRFVYLIKYANAQKIRKVAKNEKVVMFVLGGIP
UR    613  K..........GIHGLRNFITNNVIESSRFVYLIKYANAQKIREVAKNEKVVMFVLGGIP
RFF   612  KTDPVTGKPLKGKNPLRNFIANNVIENSRFIYLIKFCNPENVRKIVNNTKVTEFVLKRIP
RA    612  KTDPVTGKPLKGKNPLRNFIANNVIENSRFIYLIKFCNPENVRKIVNNTKVTEFVLKRIP
P1E0  586  TGKVRPRSDSARDTGLRNFIANNVVESRRFTYLLRYMTPEQARVLAQNEKLIAFVLSTVP
ES    616  DAN..GDKKLKKNNGLRNFIASNVIDSNRFKYLVRYGNPKKIRETAKCKPAVRFVLNEIP
RFX   578  GNK.....LKKGKHGMRNFIINNVISNKRFHYLIRYGDPAHLHEIAKNEAVVKFVLGRIA
ADM   628  KKIINKINGTKEDRNLRNFIINNVVKSRRFQYIARHMNTHYVKQLANNETLNRFVLNKMG

RSP   663  DT..........QIERYYKSCVEFPDMNSSLEVKRSELARMIKNISFDDFKNVK......
UR    663  DT..........QIERYYKSCVEFPDMNSSLGVKRSELARMIKNISFDDFKNVK......
RFF   672  DA..........QIERYYKSCTDS.EMNPPTEKKITELAGKLKDMNFGNFRNVR......
RA    672  DA..........QIERYYKSCTDS.EMNPPTEKKITELAGKLKDMNFGNFRNVR......
P1E0  646  DT..........QLERYCRTCGREDITGRPAQIR..YLTAQIMGVRYESFTDVE......
ES    674  DA..........QIERYYEACCPKNTALCSANKRREKLADMIAEIKFENFSDAGNYQKAN
RFX   633  DIQKKQGQNGKNQIDRYYETCIGK.DKGKSVSEKVDALTKIITGMNYDQFDKKR....SV
ADM   688  DAK.........IINRYYESISGN.TPNIEVRSQIDYLVKRLRSFSFEDLNDVK....QK
```

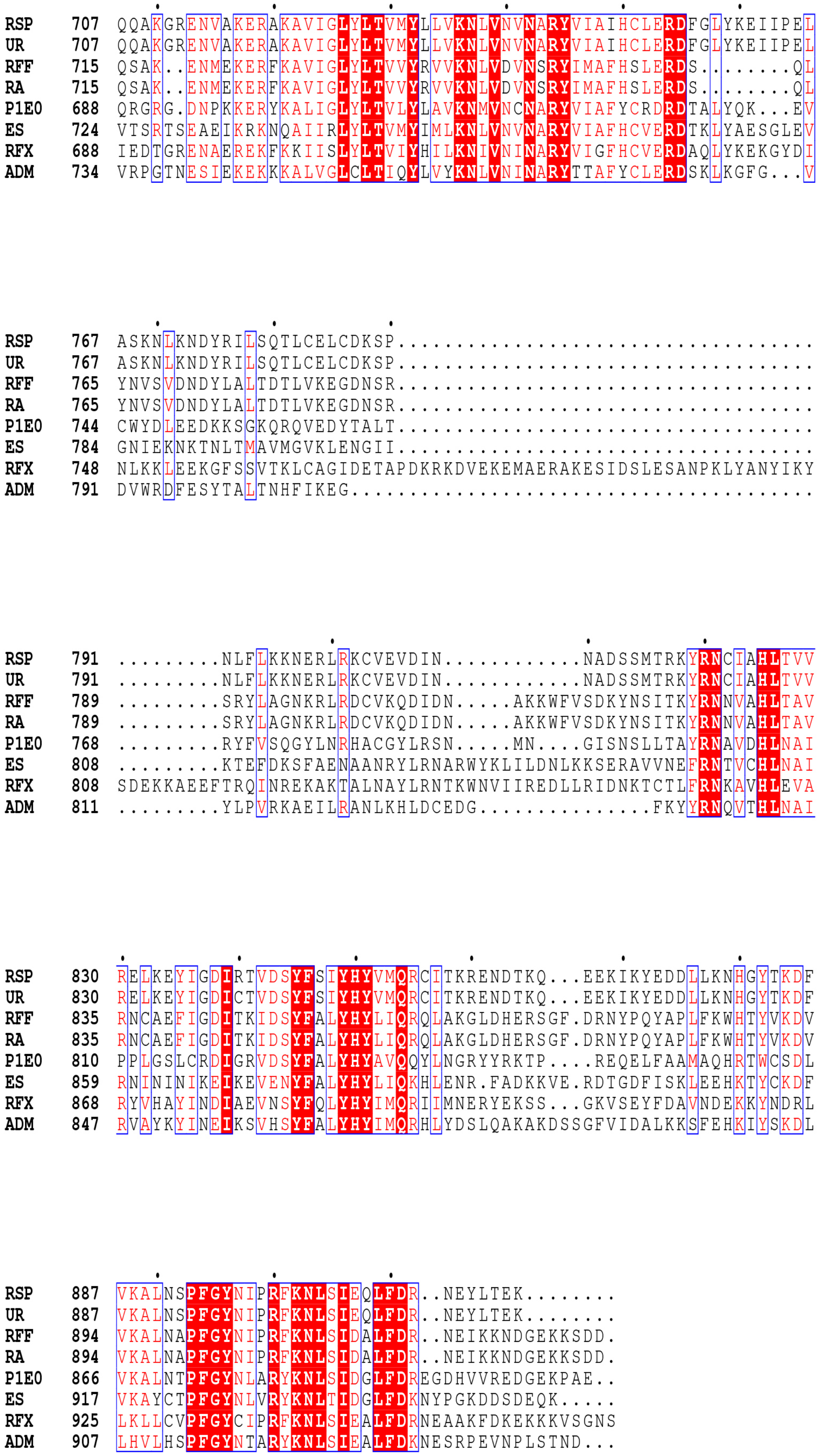

Figure S18. Multiple sequence alignment of Cas13d proteins from different species.

Sequence alignment of RspCas13d, UrCas13d, RffCas13d, RaCas13d, P1E0, EsCas13d, RfxCas13d, and ADM was performed using Clustal Omega, and the figure was prepared with ESPript (http://espript.ibcp.fr). Related to Figure S17a.

```
PBU          1  MQKQDKLFVDRKKNAIFAFPKYITIMENKEKPEPIYYELTDKHFWAAFLNLARHNVYTTI
PGU          1  .......................MTEQSERPYNGTYYTLEDKHFWAAFLNLARHNAYITL
PAU          1  ........................MEDDKKTTGSISYELKDKHFWAAFLNLARHNVYITI
PRE          1  ........................MENDKRLEESACYTLNDKHFWAAFLNLARHNVYITV
PIN          1  ........................MEDDKKTTDSIRYELKDKHFWAAFLNLARHNVYITV
RAN          1  ...........................MEKPLLPNVYTLKHKFFWGAFLNIARHNAFITI
PGI          1  ......................MNTVPASENKGQSRTVEDDPQYFGLYLNLARENLIEVE
BP           1  ......................MESIKNSQ.KSTGKTLQKDPPYFGLYLNMALLNVRKVE
CCA          1  .....................MKNIQRLGKGNEFSPFKKEDKFYFGGFLNLANNNIEDFF
BZ           1  .....................MENKTSLGNNIYYNPFKPQDKSYFAGYFNAAMENTDSVF
MOD          1  ..................MKDILTTDTTEKQNRFYSHKIADKYFFGGYFNLASNNIYEVF
PXI          1  ............................MTNTPKRRTLHRHPSYFGAFLNIARHNAFMIM
PSP          1  ...............................MNIPALVENQKKYFGTYSVMAMLNAQTVL
Alistipes    1  ..................MSNEIGAFREHQFAYAPGNEKQEEATFATYFNLALSNVEG..
FBR          1  ...................MENLNKILDKENEICISKIFNTKGIAAPITEKALDNIKSKQ

PBU         61  NHIN..RRLEIA......ELKDDGYMMGIKGSWNEQAKKLDKKVRLRDLIMKHFPFLEAA
PGU         38  THIDRQLAYSKA......DITNDQDVLSFKALWKNFDNDLERKSRLRSLILKHFSFLEGA
PAU         37  NHINKLLEIR........EIDNDEKVLDIKTLWQKGNKDLNQKARLRELMTKHFPFLETA
PRE         37  NHINKTLELKNKKNQ.EIIIDNDQDILAIKTHWAKVNGDLNKTDRLRELMIKHFPFLEAA
PIN         37  NHINKILEEG........EINRDGYETTLKNTWN.EIKDINKKDRLSKLIIKHFPFLEAA
RAN         34  CHINEQLGLKTPSN....DDKIVDVVCETWNNILNNDHDLLKKSQLTELILKHFPFLTAM
PGI         39  SHVRIKFGKK..........KLN.EESLKQSLLCDHLLSVDRWTKVYGHSRRYLPFLHYF
BP          38  NHIRKWLGDV..........ALLPEKSGFHSLLTTDNLSSAKWTRFYYKSRKFLPFLEMF
CCA         40  KEIITRFGIVITD...ENKKPKETFGEKILNEIFKKDISIVDYEKWVNIFADYFPFTKYL
BZ          40  RELG............KRLKGKEYTSENFFDAIFKENISLVEYERYVKLLSDYFPMARLL
MOD         43  EEVNKRNTFG......KLAKRDNGNLKNYIIHVFKDELSISDFEKRVAIFASYFPILETV
PXI         33  EHLSTKYDMEDKN....TLDEAQLPNAKLFGCLKKRYGKPDVTEGVSRDLRRYFPFLNYP
PSP         30  DHIQKVADIEGEQNENNENLWFHPVMSHLYNAKNGYDKQPEKTMFIIERLQSYFPFLKIM
Alistipes   41  .......................MMFGEVESNPDKIEKSLDTLPPAILRQIASFIWLSKE
FBR         42  KNDLNKEARLHYFS..IGHSFKQIDTKKVFDYVLIEELKDEKPLKFITLQKDFFTKEFSI

PBU        113  AYEMTNSKSPNN...........KEQREKEQSEALSLNNLKNVLFIFLEKLQVLRNYYSH
PGU         92  AYGKKLFESKSSGNKSSKNKELTKKEKEELQANALSLDNLKSILFDFLQKLKDFRNYYSH
PAU         89  IYTKNKED..............KKEVKQEKQAEAQSLESLKDCLFLFLDKLQEARNYYSH
PRE         96  IYSNNKED..............KEEVKEEKQAKAQSFKSLKDCLFLFLEKLQEARNYYSH
PIN         88  TYRLNPTD..............TTKQKEEKQAEAQSLESLRKSFFVFIYKLRDLRNHYSH
RAN         90  CYHPPKKEGKKKGHQ..KEQQKEKESEAQSQAEALNPSKLIEALEILVNQLHSLRNYYSH
PGI         88  DPDSQ...................IEKDHDSKTGVDPDSAQRLIRELYSLLDFLRNDFSH
BP          88  DSDKKSYE...............NRRETAECLDTIDRQKISSLLKEVYGKLQDIRNAFSH
CCA         97  SLY........................LEEMQFKNRVICFRDVMKELLKTVEALRNFYTH
BZ          88  DK..........................KEVPIKERKENFKKNFKGIIKAVRDLRNFYTH
MOD         97  DKKSIKER.................NRTIDLTLSQRIRQFREMLISLVTAVDQLRNFYTH
PXI         89  LFLHLEKQ................QNAEQAATYDINPEDIEFTLKGFFRLLNQMRNNYSH
PSP         90  AENQREYSN.............GKYKQNRVEVNSNDIFEVLKRAFGVLKMYRDLTNHYKT
Alistipes   78  DHP............................DKAYSTEEVKVIVTDLVRRLCFYRNYFSH
FBR        100  KLQKLINSIRN............INNHYVHNFNDINLNKIDSNVFHFLKESFELAIIEKY

PBU        162  YKYSEESPKPIFETSLLKNMYKVFDANVRLVKRDYMHHENIDMQRDFTHLNRKKQVGRTK
PGU        152  YRHSESSELPLFDGNMLQRLYNVFDVSVQRVKRDHEHNDKVDPHRHFNHLVRKGKKDRYG
PAU        135  YKYSEFSKEPEFEEGLLEKMYNIFGNNIQLVINDYQHNKDINPDEDFKHLDRK.......
PRE        142  YKYSESSKEPEFEEGLLEKMYNTFDASIRLVKEDYQYNKDIDPEKDFKHLERK.......
PIN        134  YKHSKSLERPKFEEGLLEKMYNIFNASIRLVKEDYQYNKDINPDEDFKHLDRT.......
RAN        148  YKHK....PDAEKDIFKHLYKAFDASLRMVKEDYKAHFTVNLTRDFAHLNRKGKNKQ..
PGI        129  NRLDGTTFEH..LEVSPDISSFITGTYSLACGRAQSRFAVFFKPDDFVLAKNRK......
BP         133  YHIDDQSVKHTALIISSEMHRFIENAYSFALQKTRARFTGVFVETDFLQAEEKG......
CCA        133  YDHEPIKIEDRVFYFLDKVLLDVSLTVKNKYLKTDKTKEFLNQHIGEELKELCKQRKDYL
BZ         122  KEHGEVEITDEIFGVLDEMLKSTVLTVKKKKVKTDKTKEILKKSIEKQLDILCQKKLEYL
MOD        140  YHHSEIVIENKVLDFLNSSLVSTALHVKDKYLKTDKTKEFLKETIAAELDILIEAYK...
PXI        133  YISNTDYGKFDKLPVQDIYEAAIFRLLDRGKHTKRFDVFESKHTRHLESNNSE.......
PSP        137  YEEKLNDGCEFLTSTEQPLSGMINNYYTVALRNMNERYGYKTEDLAFIQDKRFKFVKDAY
Alistipes  110  CFYLDTQYFYSDELVDTTAIGEKLPYNFHHFITNRLFRYSLPEITLFR............
FBR        148  YKVNKKYPLDNEIVLFLKELFIKDENTALLNYFTNLSKDEAIEYILTFTITENKIWN...

PBU        222  N...........................................................
PGU             ............................................................
PAU             ............................................................
PRE             ............................................................
PIN             ............................................................
RAN             ............................................................
PGI             ............................................................
BP              ............................................................
CCA        193  VGKGKRIDKESEIINGIYNNAFKDFICKREKQDDKENHNSVEKILCNKEPQNKKQKSSAT
BZ         182  RDTARKIEEKRRNQR...ERGEKELVAPFKYSDKRDDLIAAIYNDAFDVYIDKKKDS...
MOD        197  ...KKQIEKKN................TRFKANKREDILNAIYNEAFWSFINDKDKD...
PXI             ............................................................
PSP        197  GKKKSQVNTG..................................................
Alistipes       ............................................................
FBR             ............................................................
```

```
PBU        223  .............IIDSPNFHYHFADKEGNMTIAGLLFFVSLFLDKKDAIWMQKKLKGFK
PGU        212  .............HNDNPSFKHHFVDGEGMVTEAGLLFFVSLFLEKRDAIWMQKKIRGFK
PAU        188  ...............GQFKYSFAD..NEGNITESGLLFFVSLFLEKKDAIWMQQKLNGFK
PRE        195  ...............EDFNYLFTDKDNKGKITKNGLLFFVSLFLEKKDAIWMQQKFRGFK
PIN        187  ...............EEEFNYYFTKDNEGNITESGLLFFVSLFLEKKDAIWMQQKLRGFK
RAN        202  ...............DNPDFNRYRFEKDGFFTESGLLFFTNLFLDKRDAYWMLKKVSGFK
PGI        181  ...............EQLISVADGKE...CLTVSGFAFFICLFLDREQASGMLSRIRGFK
BP         187  ...............DNKKFFAIGGNEGIKLKDNALIFLICLFLDREEAFKFLSRATGFK
CCA        253  VWELCSKSSSKYTEKSFPNRENDKHCLEVPISQKGIVFLLSFFLNKGEIYALTSNIKGFK
BZ         236  .....LKESSKAKYNTKSDPQQEEGDLKIPISKNGVVFLLSLFLTKQEIHAFKSKIAGFK
MOD        235  ..KETVVAKGADAYFEKNHHKSNDPDFALNISEKGIVYLLSFFLTNKEMDSLKANLTGFK
PXI        186  ....................YRPRSLANSPDHENTVAFVTCLFLERKYAFPFLSRLDCFR
PSP        207  .............FFLSLQDYNGDTQKKLHLSGVGIALLICLFLDKQYINIFLSRLPIFS
Alistipes  158  .....................WNEGERKYEILRDGLIFFCCLFLKRGQAERFLNELRFFK
FBR        205  ...............INNEHNILNIEKGKYLTFEAMLFLITIFLYKNEANHLLPKLYDFK

PBU        270  DGRNLRE................QMTNEVFCRSRISLPKLKLENVQT.............
PGU        259  GGTETYQ................QMTNEVFCRSRISLPKLKLESLRT.............
PAU        231  DNLENKK................KMTHEVFCRSRILMPKLRLESTQT.............
PRE        240  DNRGNKE................KMTHEVFCRSRMLLPKIRLESTQT.............
PIN        232  DNRENKK................KMTNEVFCRSRMLLPKLRLQSTQT.............
RAN        247  ASHKQRE................KMTTEVFCRSRILLPKLRLESRYD.............
PGI        223  RTDENWA................RAVHETFCDLCIRHPHDRLESSNT.............
BP         232  STKEKGF................LAVRETFCALCCRQPHERLLSVNP.............
CCA        313  AKITKEE.....PVTYDKNSIRYMATHRMFSFLAYKGLKRKIRTSEINYNEDG..QASST
BZ         291  ATVIDEATVSEATVSHGKNSICFMATHEIFSHLAYKKLKRKVRTAEINYGEAENAEQLSV
MOD        293  GKVDRES..........GNSIKYMATQRIYSFHTYRGLKQKIRTSEE.............
PXI        226  STNDAAE..........GDPLIRKASHECYTMFCCRLPQPKLE.................
PSP        254  SYNAQSE..............ERRIIIRSFGINSIKLPKDRIHSEKS.............
Alistipes  197  RTDEEGR.................IKRTIFTKYCTRESHKHIGIEEQ.............
FBR        250  NNKSKQE.....................LFTFFSKKFTSQDIDAEEG.............

PBU        301  ..KDWMQLDMLNELVRCPKSLYERLREKDRESFKVPFDIFSDDYNAE.............
PGU        290  ..DDWMLLDMLNELVRCPKPLYDRLREDDRACFRVPVDILPDEDDTDGGG..........
PAU        262  ..QDWILLDMLNELIRCPKSLYERLQGDDREKFKVPFDPADEDYNAE.............
PRE        271  ..QDWILLDMLNELIRCPKSLYERLQGAYREKFKVPFDSIDEDYDAE.............
PIN        263  ..QDWILLDMLNELIRCPKSLYERLREEDREKFRVPIEIADEDYDAE.............
RAN        278  ..HNQMLLDMLSELSRCPKLLYEKLSEENKKHFQVEADGFLDEIEEE.............
PGI        254  ..KEALLLDMLNELNRCPRILYDMLPEEERAQFLPALDENSMNNLSENSLDEESRLLWDG
BP         263  ..REALLMDMLNELNRCPDILFEMLDEKDQKSFLPLLGEEEQAHILENSLNDELCEAIDD
CCA        366  YEKETLMLQMLDELNKVPDVVYQNLSEDVQKTFIEDWNEYLKENNGDVGTMEE.......
BZ         351  YAKETLMMQMLDELSKVPDVVYQNLSEDVQKTFIEDWNEYLKENNGDVGTMEE.......
MOD        330  GVKETLLMQMIDELSKVPNVVYQHLSTTQQNSFIEDWNEYYKDYEDDVETDDL.......
PXI        259  ..SSDILLDMVNELGRCPSALYNLLSEEDQARFHIKREEITGFEEDPD............
PSP        287  ..NKSVAMDMLNEVKRCPDELFTTLSAEKQSRFRIISDDHN...................
Alistipes  227  ..DFLIFQDIIGDLNRVPKVCDGVVDLSKENERYIKNRETSNESDEN.............
FBR        276  ..HLIKFRDMIQYLNHYPTAWNNDLKLESENKNKIMTTKLIDSIIEFELNSN........

PBU        346  ...EEPFKNTLVRHQDRFPYFVLRYFDLNEIFEQLRFQIDLG.TYHFSIYNKRIGDE.DE
PGU        338  ...EDPFKNTLVRHQDRFPYFALRYFDLKKVFTSLRFHIDLG.TYHFAIYKKMIGEQ.PE
PAU        307  ...QEPFKNTLIRHQDRFPYFVLRYFDYNEIFKNLRFQIDLG.TYHFSIYKKLIGGQ.KE
PRE        316  ...QEPFRNTLVRHQDRFPYFALRYFDYNEIFKNLRFQIDLG.TYHFSIYKKLIGGK.KE
PIN        308  ...QEPFKNTLVRHQDRFPYFALRYFDYNEIFTNLRFQIDLG.TYHFSIYKKQIGDY.KE
RAN        323  ...QNPFKDTLIRHQDRFPYFALRYLDLNESFKSIRFQVDLG.TYHYCIYDKKIGDE.QE
PGI        312  SSDWAEALTKRIRHQDRFPYLMLRFIEEMDLLKGIRFRVDLG.EIELDSYSKKVGRNGEY
BP         321  PFEMIASLSKRVRYKNRFPYLMLRYIEEKNLLPFIRFRIDLG.CLELASYPKKMGEENNY
CCA        419  ..EQVIHPVIRKRYEDKFNYFAIRFLDEFAQFPTLRFQVHLG.NYLCDKR.TKQICDTTT
BZ         404  ..EQVIHPVIRKRYEDKFNYFAIRFLDEFAQFPTLRFQVHLG.NYLHDSR.PKENLIS..
MOD        383  ..SRVIHPVIRKRYEDRFNYFAIRFLDEFFDFPTLRFQVHLG.DYVHDRR.TKQLGKVES
PXI        305  ..EELEQEIVLKRHSDRFPYFALRYFDDTEAFQTLRFDVYLG.RWRTKPVYKKRIYGQER
PSP        326  .......EVLMKRSSDRFVPLLQYIDYGKLFDHIRFHVNMGKLRYLLKADKTCIDGQTR
Alistipes  272  ....KARYRLLIREKDKFPYYLMRYIVDFGVLPCITFKQNDYSTKEGRGQFHYQDAAVAQ
FBR        326  ..YPSFATDIQFKKEAKAFLFASNKKRNQTSFSNKSYNEEIRHNPHIKQYRDEIASALTP

PBU        401  VRHLTHHLYGFARIQDFAPQNQPEEWRKLVKDLDHFETSQEPYISKTAPHYHLENEKIGI
PGU        393  DRHLTRNLYGFGRIQDFAEEHRPEEWKRLVRDLDYFETGDKPYISQTSPHYHIEKGKIGL
PAU        362  DRHLTHKLYGFERIQEFAKQNRPDEWKAIVKDLDTYETSNKRYISETTPHYHLENQKIGI
PRE        371  DRHLTHKLYGFERIQEFTKQNRPDKWQAIIKDLDTYETSNERYISETTPHYHLENQKIGI
PIN        363  SHHLTHKLYGFERIQEFTKQNRPDEWRKFVKTFNSFETSKEPYIPETTPHYHLENQKIGI
RAN        378  KRHLTRTLLSFGRLQDFTEINRPQEWKALTKDLDYKETSNQPFISKTTPHYHITDNKIGF
PGI        371  DRTITDHALAFGKLSDFQNEEE.......VSRMISGEASYPVRFSLFAPRYAIYDNKIGY
BP         380  ERSVTDHAMAFGRLTDFHNEDA.......VLQQITKGITDEVRFSLYAPRYAIYNNKIGF
CCA        475  EREVKKKITVFGRLSELENKKAIFLN...EREE..IKG....WEVFPNPSYDFPKENISV
BZ         458  DRRIKEKITVFGRLSELEHKKALFIK...NTET..NEDREHYWEIFPNPNYDFPKENISV
MOD        439  DRIIKEKVTVFARLKDINSAKASYFH...SLEEQDKEELDNKWTLFPNPSYDFPKEHT..
PXI        362  DRVLTQSIRTFTRLSRLLPIYENVKHDAVRQNEEDGKLVNPDVTSQFHKSWIQIESDDRA
PSP        379  VRVIEQPLNGFGRLEEAETMRKQENGTFGNSGIRIRDFENMKRDDANPANYPYIVDTYTH
Alistipes  328  EERCYNFVVRNGNVYYS.............YMPQAQNVVRISELQGTISVEELRNMVYAS
FBR        384  ISFNVKEDKFKIFVKKHVLEEYFPNSIGYEKFLEYNDFTEKEKEDFGLKLYSNPKTNKLI
```

```
PBU        461  KFCSAHNNLFPSLQTDKTCNGRSKFNLGTQFTAEAFLSVHELLPMMFYYLLLTKDYSRK.
PGU        453  RFMPEGQHLWPSPEVGTTRTGRSKYAQDKRLTAEAFLSVHELMPMMFYYFLLREKYSEE.
PAU        422  RFRNGNKEIWPSLKTNDENNEKSKYKLDKQYQAEAFLSVHELLPMMFYYLLLKKEKPNND
PRE        431  RFRNDNNDIWPSLKTNGEKNEKSKYNLDKPYQAEAFLSVHELLPMMFYYLLLKMENTDND
PIN        423  RFRNDNDKIWPSLKTNSEKNEKSKYKLDKSFQAEAFLSVHELLPMMFYYLLLKTENTDND
RAN        438  RLG.TSKELYPSLEIKDGANRIAKYPYNSGFVAHAFISVHELLPLMFYQHLTG.......
PGI        424  CHTSDPVYPKSKTGEKR.........ALSNPQSMGFISVHDLRKLLLMELLCEGSFSRMQ
BP         433  VRTSGSDKISFPTLKKKGGEGHCVAYTLQNTKSFGFISIYDLRKILLLSFLDKDKAKNIV
CCA        526  NYKDFPIVGSILDREKQPVSNKIGIRVKIADELQR.EIDKAIKEKKLRNPKNRKANQDEK
BZ         513  NDKDFPIAGSILDREKQPVAGKIGIKVKLLNQQYVSEVDKAVKAHQLKQRKASKPSIQN.
MOD        494  .........LQHQGEQKNAGKIGIYVKLRDTQYK...EKAALEEARKSLNPKERSATKA
PXI        422  FLSDRIEHFSPHYNFGDQVIGLKFINPDRYAAIQNVFPKLPGEEKKDKDAKLVNETADAI
PSP        439  YILENNKVEMFINDKEDSAPLLPVIEDDRYVVKTIPSCRMSTLEIPAMAFHMFLFGSKK.
Alistipes  375  INGKDVNKSVEQYLYHLHLLYEKILTISGQTIKEGRVDVEDYRPLLDKLLLRPASNGEE.
FBR        444  ERIDNHKLVKSHGRNQDRFMDFSMRFLAENNYFGKDAFFKCYKFYDTQEQDEFLQSNENN
```

```
PBU        520  .......................ESADKVEGIIRKEISNIYAIYDAFANNEINSIADLT
PGU        512  .......................VSAEKVQGRIKRVIEDVYAIYDAFARDEINTLKELD
PAU        482  EIN.......................ASIVEGFIKREIRNIFKLYDAFANGEINNIDDLE
PRE        491  KEDNEVGTKKKGNKNNK.......QEKHKIEEIIENKIKDIYALYDAFTNGEINSIDELA
PIN        483  NEIETK...KKENKNDK.......QEKHKIEEIIENKITEIYALYDTFANGEIKSIDELE
RAN        490  ............................KSEDLLKETVRHIQRIYKDFEEERINTIEDLE
PGI        475  SDFLRKANRILDETAEG.....KLQFSALFPEMRHRFIPPQNPKSKDRREKAETTLEKYK
BP         493  SGLLEQCEKHWKDLSENLFDAIRTELQKEFPVPLIRYTLPRSKGGKLVSSKLADKQEKYE
CCA        585  QKE...................RLVNEIVSTNSNEQGE.PVVFIGQPTAYLSMNDIHS
BZ         572  .......................IIEEIVPINESNPKE.AIVFGGQPTAYLSMNDIHS
MOD        541  SKY...................DIITQIIEANDNVKSEKPLVFTGQPIAYLSMNDIHS
PXI        482  ISTHEIR...........SLFLYHYLSKKPISAGDERRFIQVDTETFIKQYIDTIKLFF
PSP        498  ...........................TEKLIVDVHNRYKRLFQAMQKEEVTAENIAS
Alistipes  434  .................................LRRELRKLLPKRVCDLLSNRFDCS
FBR        504  DD....................VKFHKGKVTTYIKYEEHLKNYSYWDCPFVEENNSMS
```

```
PBU        556  RRLQNTNILQGHLPKQMISILKGRQKDMGKEAERKIGEMIDDTQRRLDLLCKQTNQKI.R
PGU        548  ACLADKGIRRGHLPKQMIAILSQEHKDMEEKIRKKLQEMIADTDHRLDMLDRQTDRKI.R
PAU        519  KYCADKGIPKRHLPKQMVAILYDEHKDMVKEAKRKQKEMVKDTKKLLATLEKQTQKEK.E
PRE        544  EQREGKDIEIGHLPKQLIVILKNKSKDMAEKANRKQKEMIKDTKKRLATLDKQVKGEI.E
PIN        533  EYCKGKDIEIGHLPKQMIAILKDEHKVMATEAERKQEEMLVDVQKSLESLDNQINEEI.E
RAN        522  KANQGR.LPLGAFPKQMLGLLQNKQPDLSEKAKIKIEKLIAETKLLSHRLNTKLKSSP.K
PGI        530  QEIKGRKDKLNSQLLSAFDMDQRQLPSRLLDEWMNIRPASHSVKLRTYVKQLNEDCRL.R
BP         553  SEFERRKEKLT.EILSEKDFDLSQIPRRMIDEWLNVLPTSREKKLKGYVETLKLDCRE.R
CCA        623  VLYEFLINKIS................GEALETKIVEKIETQIKQIIGKDATTKILK.P
BZ         606  ILYEFFDKWEKKKEKLEKKGEKELRKEIGKELEKKIVGKIQAQIQQIIDKDTNAKILK.P
MOD        580  MLFSLLTDNAELKKTP............EEVEAKLIDQIGKQINEILSKDTDTKILK.K
PXI        530  EDIKSGELQPIADPPNYQKNEPLPYVRGDKEKTQEERAQYRERQKEIKERRKELNTLL.Q
PSP        529  FGIAESDLPQKILDLISGNAHGKDVDAFIRLTVDDMLTDTERRIKRFKDDRKSIRSADNK
Alistipes  458  EGVSAVEKRLKAILLRHEQLLLSQNPALHIDKIKSVIDYLYLFFSDDEKFRQQPTEKAHR
FBR        542  VKISIGSEEKILKIQRNLMIYFLENALYNENVENQGYKLVNNYYRELKKDVEESIASLDL
```

```
PBU        615  IGKRNAGLLKSGKIADWLVNDMMRFQPVQKDQNNIPINNSKANSTEYR............
PGU        607  IGRKNAGLPKSGVIADWLVRDMMRFQPVAKDTSGKPLNNSKANSTEYR............
PAU        578  DDGRNVKLLKSGEIARWLVNDMMRFQPVQKDNEGKPLNNSKANSTEYQ............
PRE        603  DGGRNIRLLKSGEIARWLVNDMMRFQPVQKDNEGKPLNNSKANSTEYQ............
PIN        592  NVERKNSSLKSGKIASWLVNDMMRFQPVQKDNEGKPLNNSKANSTEYQ............
RAN        580  LGKRREKLIKTGVLADWLVKDFMRFQPVAYDAQNQPIKSSKANSTEFW............
PGI        589  LRKFRKDGDGKARAIPLVGEMATFLSQDIVRMIISEETKKLITSAYYN............
BP         611  LRVFEKREKGEHPLPPRIGEMATDLAKDIIRMVIDQGVKQRITSAYYS............
CCA        665  YTNANSNSINREKLLRDLEQEQQILKTLLEEQQQREKDKKDKKSK...............
BZ         665  YQDGNSTAIDKEKLIKDLKQEQNILQKLKDEQTVREKEYNDFIAYQDKNREINKVRDRNH
MOD        626  YKDNDLKETDTDKITRDLARDKEEIEKLILEQKQRADDYNYTSSTKFN............
PXI        589  NRYGLSIQYIPSRLREYLLGYKKVPYEKLALQKLRAQRKEVKKRIKDIEKMR........
PSP        589  MGKRGFKQISTGKLADFLAKDIVLFQPSVNDGENKITGLNYRIMQSAI............
Alistipes  518  GLKDEEFQMYHYLVGDYDSHPLALWKELEASGRLKPEMRKLTSATSLHG...........
FBR        602  IKSNPDFKSKYKKILPKRLLHNYAPAKQDKAPENAFETLLKKADFREEQ...........
```

```
PBU        663  .............MLQRALALFGSENFR..LKAYFNQMNLVGNDNPHPFLAETQWEHQT
PGU        655  .............MLQRALALFGGEKKR..LTPYFRQMNLTGGNNPHPFLHETRWESHT
PAU        626  .............MLQRSLALYNNEEK...PTRYFRQVNLIESNNPHPFLKWTKWEECN
PRE        651  .............MLQRSLALYNKEEK...PTRYFRQVNLIKSSNPHPFLEDTKWEECY
PIN        640  .............LLQRTLAFFGSEHER..LAPYFKQTKLIESSNPHPFLKDTEWEKCN
RAN        628  .............FIRRALALYGGEKNR..LEGYFKQTNLIGNTNPHPFLNKFNWKACR
PGI        637  .............EMQRSLAQYAGEENRRQFRAIVAELRLLDPSSGHPFLSATMETAHR
BP         659  .............EIQRCLAQYAGDDNRRHLDSIIRELRLKDTKNGHPFLGKVLRPGLG
CCA        710  ...............RKHELYPSEKGKVAVWLANDIKRFMPKAFKEQWRGYHHSLLQK
BZ         725  KQYLKDNLKRKYPEAPARKEVLYYREKGKVAVWLANDIKRFMPTDFKNEWKGEQHSLLQK
MOD        674  ..........IDKSRKRKHLLFNAEKGKIGVWLANDIKRFMFKESKSKWKGYQHTELQK
PXI        641  ............TPRVGEQATWLAEDIVFLTPPKMHTPERKTTKHPQKLNNDQFRIMQS
PSP        637  .............AVYDSGDDYEAKQQFKLMFEKARLIGKGTTEPHPFLYKVFARSIPA
Alistipes  567  ..............LYMLCLKGTVEWCRKQLMSIGKGTAKVEAIADRVGLKLYDKLKEY
FBR        651  ..........YKKLLKKAEHEKNKEDFVKRNKGKQFKLHFIRKACQMMYFKEKYNTLKE
```

```
PBU        707  NILSFYRNYLEARKKYLKGLKPQ...........NWKQYQHFLILKVQKTNRNTLVTGWK
PGU        699  NILSFYRSYLRARKAFLERIGRS...........DRMENRPFLLLKEPKTDRQTLVAGWK
PAU        669  NILTFYYSYLTKKIEFLNKLKPE...........DWKKNQYFLKLKEPKTNRETLVQGWK
PRE        694  NILSFYRNYLKAKIKFLNKLKPE...........DWKKNQYFLMLKEPKTNRKTLVQGWK
PIN        684  NILSFYRSYLEAKKNFLESLKPE...........DWEKNQYFLKLKEPKTKPKTLVQGWK
RAN        672  NLVDFYQQYLEQREKFLEAIKNQ...........PWEPYQYCLLLKIPKENRKNLVKGWE
PGI        683  YTEGFYKCYLEKKREWLAKIFYRPE........QDENTKRRISVFFVPDGEARKLLPLLI
BP         705  HTEKLYQRYFEEKKEWLEATFYP............AASPKRVPRFVNPPTGKQKELPLII
CCA        753  YLAYYEQSKEELKNLLPKEVFKHFP..FKLKGYFQQQYLNQFYTDYLKRRLSYVNELLLN
BZ         785  SLAYYEQCKEELKNLLPEKVFQHLP..FKLGGYFQQKYLYQFYTCYLDKRLEYISGLVQQ
MOD        723  LFAYFDTSKSDLELILSDMVMVKDYPIELIDLVRKSRTLVDFLNKYLEARLGYIENVITR
PXI        688  SLAYFSVNKKAIKKFFQKETGIGLSNRETSHPFLYRIDVGRCRGILDFYTGYLKYKMDWL
PSP        683  NAVEFYERYLIERKFYLTGLSNEIK............KGNRVDVPFIRRDQNKWKTPAMK
Alistipes  612  TPEQLEREVKLVVMHGYAAAATP.............KPKAQAAIPSKLTELRFYSFLGKR
FBR        700  GNAAFEKKDPVIEKRKNKEHEFGHHKN......LNITREEFNDYCKWMFAFNGNDSYKKY

PBU        756  N.SFNLPRGIFTQPIREWFEKHNNSKR.IYDQILSFDRVG.........FVAKAIPLYFA
PGU        748  S.EFHLPRGIFTEAVRDCLIEMG......YDEVGSYREVG.........FMAKAVPLYFE
PAU        718  N.GFNLPRGIFTEPIREWFKRHQNNSK.EYEKVEALDRVG.........LVTKVIPLFFK
PRE        743  N.GFNLPRGIFTEPIKEWFKRHQNDSE.EYKKVEALDRVG.........LVAKVIPLFFK
PIN        733  N.GFNLPRGIFTEPIRKWFMKHREN.....ITVAELKRVG.........LVAKVIPLFFS
RAN        721  QGGISLPRGLFTEAIRETLSEDLMLSKPIRKEIKKHGRVG.........FISRAITLYFK
PGI        735  RRRMKEQNDLQDWIRNKQAHPIDLPSHLFDSKVMELLKVKDGKK.....KWNEAFKDWWS
BP         753  RNLMKERPEWRDWKQRKNSHPIDLPSQLFENEICRLLKDKIGKEPSGKLKWNEMFKLYWD
CCA        811  IQNFKNDKDALKATEKECFKFFRKQNYIINPINIQIQSILVYPIFLKRGFLDEKPTMIDR
BZ         843  AENFKSENKVFKKVENECFKFLKKQNYTHKELDARVQSILGYPIFLERGFMDEKPTIIKG
MOD        783  VKNSIG.TPQFKTVRKECFAFLKESNYTVASLDKQIERILSMPLFIERGFMDSKPTMLEG
PXI        748  DDAIKKVDNRKHGKKEAKKYEKYLPSSIQHKTPLELDYTRLPVYLPRGLFKKAIVKALAA
PSP        731  TLGRIYSEDLPVELPRQMFDNEIKSHLKSLPQMEGIDFNNANVTYLIAEYMKRVLDDDFQ
Alistipes  659  EMSFAAFIRQDKKAQKLWLRNFYTVENIKTLQKRQ.........................
FBR        754  LRDLFSEKHFFDNQEYKNLFESSVNLEAFYAKTKELFKKWIETNKPTNNENRYTLENYKN

PBU        805  EEYKDN................VQPFYDYPFNIGNRLKPKKRQFLDKKERVELWQKNKEL
PGU        792  RACEDR................VQPFYDSPFNVGNSLKPKKGRFLSKEERAEEWERGKER
PAU        767  EEYFKDKEENFKEDTQKEINDCVQPFYNFPYNVGNIHKPKEKDFLHREERIELWDKKKDK
PRE        792  EEYFK.......EDAQKEINNCVQPFYSFPYNVGNIHKPEEKNFLHCEERRKLWDKKKDK
PIN        778  EEYKDS................VQPFYNYHFNVGNINKPDEKNFLNCEERRELLRKKKDE
RAN        772  EKYQDK................HQSFYNLSYKLEAKAP.....LLKREEHYEYWQQNKPQ
PGI        790  TKYPDG................MQPFYGLRRELNIHGKSVSYIPSD.GKKFADCYTHLME
BP         813  KEFPNG................MQRFYRCKRRVEVFDKVVEYEYSEEGGNYKKYYEALID
CCA        871  EKFKENK...............DTELADWFMHYKNYK.EDNYQKFYAYPLEKVEEKEK.F
BZ         903  KTFKGN................EALFADWFRYYKEYQNFQTFYDTENYPLVELEKKQADR
MOD        842  KSYQQH................KEDFADWFVHYKENSNYQNFYDTEVYEIITEDKREQ.A
PXI        808  HADFQVE..............PEEDNVIFCLDQLLDGDTQDFYNWQRYYRSALTEKETDN
PSP        791  TFYQWNR...............NYRYMDMLKGEYDRKGSLQHCFTSVEEREGLWKERASR
Alistipes  694  .......................AAADAACKKLYNLVGEVERVHTNDKVLVLVAQRYRER
FBR        814  LILQKQVFIN............VYHFSKYLIDKNLLNSENNVIQYKSLENVEYLISDFYF

PBU        849  FKNYPSEKK...................................................
PGU        836  FRDLEAWSHSAARRIEDAFAGIEYASPGNKKKIEQLLRDLSLWEAFESKLKVRADKINLA
PAU        827  FKGYKEKIKSKKLTEKD...........................................
PRE        845  FKGYKAKEKSKKMTDKE...........................................
PIN        822  FK.........KMTDKE...........................................
RAN        811  SPTESQRLELHTS...............................................
PGI        833  KTVRD.......................................................
BP         857  EVVRQ.......................................................
CCA        914  KRNKQIN.....................................................
BZ         947  KRKTKIY.....................................................
MOD        885  KVTKKIK.....................................................
PXI        854  QLVLAHP.....................................................
PSP        836  TERYRKQASN..................................................
Alistipes  731  LLNVG.......................................................
FBR        862  QSKLSIDQYKTCG...............................................

PBU        858  .........KTDLAYLDFLSWKKFERELRLIKNQDIVTWLMFKELFN.MATVEGLKIGEIH
PGU        896  KLKKEILEAQEHPYHDFKSWQKFERELRLVKNQDIITWMMCRDLME.ENKVEGLDTGTLY
PAU        844  .......KEEFRSYLEFQSWNKFERELRLVRNQDIVTWLLCKELID.KLKIDELNIEELK
PRE        862  .......KEEHRSYLEFQSWNKFERELRLVRNQDILTWLLCTKLID.KLKIDELNIEELQ
PIN        830  .......KEENPSYLEFKSWNKFERELRLVRNQDIVTWLLCMELFN.KKKIKELNVEKIY
RAN        824  ........DRWKDYLLYKRWQHLEKKLRLYRNQDVMLWLMTLELTKNHFKELNLNYHQLK
PGI        838  .......KKRELRTAGKPVPPDLAADIKRSFHRAVNEREFMLRLVQEDDRLMLMAINKMM
BP         862  .......KISSSKEKSKLQVEDLTLSVRRVFKRAINEKEYQLRLLCEDDRLLFMAVRDLY
CCA        921  .......KQKKNDVYTLMMVEYIIQKIFGDKFVEENPLVLKGIFQSKAERQQNNTHAATT
BZ         954  .......QQKKNDVFTLLMAKHIFKSVFKQDSID..QFSLEDLYQSREERLGNQERARQT
MOD        892  .......QQQKNDVFTLMMVNYMLEEVLKLPSND..RLSLNELYQTKEERIVNKQVAKDT
PXI        861  ......YAEQILGTIKTLEGKQKNNKLGNKAKQKIKDELIDLKRAKRRLLDREQYLRAVQ
PSP        846  .......KIRSNRQMRNASSEEIETILDKRLSNSRNEYQKSEKVIRRYRVQDALLFLLAK
Alistipes  736  ........SKCAVTLDNPERQQKLADVYEVQNAWLSIRFDDLDFTLTHVNLSNLRKAYNL
FBR        875  ........KLFNKLKSNKLEDCLLYEIAYNYIDKKNVHKIDIQKILTSKIILTINDANTP
```

```
PBU         909  LRDIDTNTANEES..............NNILNRIMPMKLPVKTYETDNKGNILKERP..
PGU         955  LKDIRPNVQEQGS..............LNVLNRVKPMRLPVVVYRADSRGHVHKEEAP.
PAU         896  KLRLNNIDTDTAK...........KEKNNILNRVMPMELPVTVYEIDDSHKIVKDKP..
PRE         914  KLRLKDIDTDTAK...........KEKNNILNRVMPMRLPVTVYEIDKSFNIVKDKP..
PIN         882  LKNINTNTTKKEKNTEEKNGEEKNIKEKNNILNRIMPMRLPIKVYGRENFSKNKKKKIRR
RAN         876  LENLAVNVQEADAK.............LNPLNQTLPMVLPVKVYPATAFGEVQYHKTP.
PGI         891  TDREEDILP.................GLKNIDSILDEENQFSLAVHAKVLEKEGEGGDN
BP          915  DWKEAQLD...................LDKIDNMLGEPVSVSQVIQLEGGQPD......
CCA         974  QERNLNGILNQPKD......IKIQG.KITVKGVKLKDIGNFRKYEIDQRVNTFLDYEPR
BZ         1005  GERNTNYIWNKTVD.......LKLCDGKITVENVKLKNVGDFIKYEYDQRVQAFLKYEEN
MOD         943  QERNKNYIWNKVVD.......LQLCEGLVRIDKVKLKDIGNFRKYENDSRVKEFLTYQSD
PXI         915  AEDRALWLMIQERQ..............KQKAEHEEIAFDQLDLKNITKILTESIDARL
PSP         899  KTLTELADFDGER...............FKLKEIMPDAEKGILSEIMPMSFTFEKGGK.
Alistipes   788  IPR..........................KHILAFKEYLDN..........
FBR         927  YKISVPFNKLERYT...........EMIAIKNQNNLKARFLIDLPLYLSKNKIKKGKDS

PBU         952  ..LATFYIEETETKVLKQGNFKALVKDRRLNGLFSFAETT.DLNLEEHPISKLSVDLELI
PGU         999  ..LATVYIEERDTKLLKQGNFKSFVKDRRLNGLFSFVDTG.GLAMEQYPISKLRVEYELA
PAU         942  ..LHTIYIKEAETKLLKQGNFKALVKDRRLNGLFSFVKTNSEAESKRNPISKLRVEYELG
PRE         960  ..LHTVYIEETGTKLLKQGNFKALVKDRRLNGLFSFVKTSSEAESKSKPISKLRVEYELG
PIN         942  NTFFTVYIEEKGTKLLKQGNFKALERDRRLGGLFSFVKTPSKAESKSNTISKLRVEYELG
RAN         921  ..IRTVYIREEHTKALKMGNFKALVKDRRLNGLFSFIKEE..NDTQKHPISQLRLRRELE
PGI         933  SLSLVPATIEIKSKRKDWSKYIRYRYDRRVPGLMSHFPEH........KATLDEVKTLLG
BP          949  ......AVIKAECKLKDVSKLMRYCYDGRVKGLMPYFANH........EATQEQVEMELR
CCA        1026  KEWMAYLPNDWKEKEKQGQLPPNNVIDRQISKYETVRSKILLKDVQELEKIISDEIKEEH
BZ         1058  IEWQAFLIKESKEEENYP.....YVVEREIEQYEKVRREELLKEVHLIEEYILEKVKDKE
MOD         996  IVWSGYLSNEVDSN.......KLYVIERQLDNYESIRSKELLKEVQEIECIVYNQVANKE
PXI         960  RIPDTKVDITDKLPLRRYGDLRRVAKDRRLVNLASYYHVAG.....LSEIPYDLVKKELE
PSP         942  ....KYTITSEGMKLKNYGDFFVLASDKRIGNLLELVGSD........IVSKEDIMEEFN
Alistipes   803  ...RVKQKLCEECRNVRRKEDLCTCCSPRYSNLTSWLKEN.......HSESSIEREAATMM
FBR         975  AGYEIIIKNDLEIEDINTINNKIINDSVKFTEVLMELEKYFILKDKCILSKNYIDNSEIP

PBU        1009  KYQTTRISIFEMTLGLEEKKLIDKYSTLP..TDSFR.....NMLERWLQ..CKANRPELKN
PGU        1056  KYQTARVCVFELTLRLEESLLTRYPHLP..DESFR.....KMLESWSDP.LLAKWPELHG
PAU        1000  EYQEARIEIIQDMLALEEKLINKYKDLP..TNKFS.....EMLNSWLEGKDEADKARFQN
PRE        1018  AYQKARIDIIKDMLALEKTLIDNDENLP..TNKFS.....DMLKSWLKGKGEANKARLQN
PIN        1002  EYQKARIEIIKDMLALEKTLIDKYNSLD..TDNFN.....KMLTDWLELKGEPDKASFQN
RAN         977  IYQSLRVDAFKETLSLEEKLLNKHTSLSSLENEFR.....ALLEEWKKEYAASS.MVTDE
PGI         985  EYDRCRIKIFDWAFALEGAIMSDRDLKPYLHESSSREGKSGEHSTLVKMLVEKKGCLTPD
BP          995  HYEDHRRRVFNWVFALEKSVLKNEKLRRFYEES.....QGGCEHRRCIDALRKASLVSEE
CCA        1086  RHDLKQGKYYNFKYYILNGLLRQLKNENVENYKVFK..LNTNPEKVNITQLKQEATDLEQ
BZ         1113  ..ILKKGDNQNFKYYILNGLLKQLKNEDVESYKVFN..LNTEPEDVNINQLKQEATDLEQ
MOD        1049  ..SLKQSGNENFKQYVLQGLLPRG..TDVREMLILS..TDVKFKKEEIMQLGQVR.EVEQ
PXI        1015  EYDRRRVAFFEHVYQFEKEVYDRYAAELRNENPKGESTYFSHWEYVAVAVKHSADTHFNE
PSP         990  KYDQCRPEISSIVFNLEKWAFDTYPELSARVDREE.....KVDFKSILKILLNNKNINK
Alistipes   854  LLDVERKLLSFLLDERRKAIIEYGKFIP......................FSALVKECR
FBR        1035  SLKQFSKVWIKENENEIINYRNIACHFHLPLLETFDNLLLNVEQKFIKEELQNVSTINDL

PBU        1060  YV..NSLIAVRNAFSHNQYPMYDATLFAEVKKFT...............LFPSVDTKKI
PGU        1108  KV..RLLIAVRNAFSHNQYPMYDEAVFSSIRKYDP..............SSPDAIEERM
PAU        1053  DV..DFLIAVRNAFSHNQYPMHNKIEFANIKPFS...............LYTANNSEEK
PRE        1071  DV..GLLVAVRNAFSHNQYPMYNSEVFKGMKLLS...............LSSDIP.EKE
PIN        1055  DV..DLLIAVRNAFSHNQYPMRNRIAFANINPFS...............LSSANTSEEK
RAN        1031  HI..AFIASVRNAFCHNQYPFYKEALHAPIPLFT...............VAQPTTEEKD
PGI        1045  ES..QYLILIRNKAAHNQFPCAAEMPLIYRDVSAKVGSIEGSSAKDLPEGSSLVDSLWKK
BP         1050  EY..EFLVHIRNKSAHNQFPDLEIGKLPPNVTSG.................FCECIWSK
CCA        1144  KA..FVLTYIRNKFAHNQLPKKEFWDYCQEKYGK.....................IEK
BZ         1169  KA..FVLTYIRNKFAHNQLPKKEFWDYCQEKYGK.....................IEK
MOD        1102  DL..YSLIYIRNKFAHNQLPIKEFFDFCENNYRP.....................ISD
PXI        1075  LFK.EKVMQLRNKFHHNEFPYFDWLLPEVEKASA......................
PSP        1044  EQS.DILRKIRNAFDHNNYPDKGVVEIKALPEIA......................
Alistipes   891  LAD.AGLCGIRNDVLHDNVISYADAIGKLSAYFP.....................KEASE
FBR        1095  SKPQEYLILLFIKFKHNNFYLNLFNKNESKTIKN.....................DK

PBU        1102  ELNIAPQLLEIVGKAIKEIEKSENKN.......
PGU        1151  GLNIAHRLSEEVKQAKETVERIIQV........
PAU        1095  GLGIANQLKDKTKETTDKIKKIEKPIETKE...
PRE        1112  GLGIAKQLKDKIKETIERIIEIEKEIRN.....
PIN        1097  GLGIANQLKDKTHKTIEKIIEIEKPIETKE...
RAN        1073  GLGIAEALLKVLREYCEIVKSQI..........
PGI        1103  YEMIIRKILPILDPENRFFGKLLNNMSQPINDL
BP         1090  YKAIICRIIPFIDPERRFFGKLLEQK.......
CCA        1179  EKTYAEYFAEVFKREKEALIK............
BZ         1204  EKTYAEYFAEVFKKEKEALIK............
MOD        1137  NEYYAEYYMEIFRSIKEKYAS............
PXI        1108  .ALYADRVFDVAEGYYQKMRKLMRQ........
PSP        1077  .MSIKKAFGEYAIMK..................
Alistipes   929  AVEYIRRTKEVREQRREELMANSSQ........
FBR        1131  EVKKNRVLQKFINQVILKKK.............
```

Figure S19. Multiple sequence alignment of Cas13b proteins from different species.

Sequence alignment of PguCas13b, PauCas13b, PreCas13b, PinCas13b, RanCas13b, PgiCas13b, BpCas13b, CcaCas13b, BzoCas13b, ModCas13b, PxiCas13b, PspCas13b, Alistipes, and Fbr was

performed using Clustal Omega, and the figure was prepared with ESPript (http://espript.ibcp.fr). Related to **Figure S17b.**

Supplementary Table 1. Cas13 protein sequences used in this study.

Name	Protein sequence
RfxCas13d	MIEKKKSFAKGMGVKSTLVSGSKVYMTTFAEGSDARLEKIVEGDSIRSVNEGEAFSAE<br>MADKNAGYKIGNAKFSHPKGYAVVANNPLYTGPVQQDMLGLKETLEKRYFGESADG<br>NDNICIQVIHNILDIEKILAEYITNAAYAVNNISGLDKDIIGFGKFSTVYTYDEFKDPEHH<br>RAAFNNNDKLINAIKAQYDEFDNFLDNPRLGYFGQAFFSKEGRNYIINYGNECYDILAL<br>LSGLRHWVVHNNEEESRISRTWLYNLDKNLDNEYISTLNYLYDRITNELTNSFSKNSA<br>ANVNYIAETLGINPAEFAEQYFRFSIMKEQKNLGFNITKLREVMLDRKDMSEIRKNHK<br>VFDSIRTKVYTMMDFVIYRYYIEEDAKVAAANKSLPDNEKSLSEKDIFVINLRGSFNDD<br>QKDALYYDEANRIWRKLENIMHNIKEFRGNKTREYKKKDAPRLPRILPAGRDVSAFSK<br>LMYALTMFLDGKEINDLLTTLINKFDNIQSFLKVMPLIGVNAKFVEEYAFFKDSAKIAD<br>ELRLIKSFARMGEPIADARRAMYIDAIRILGTNLSYDELKALADTFSLDENGNKLKKGK<br>HGMRNFIINNVISNKRFHYLIRYGDPAHLHEIAKNEAVVKFVLGRIADIQKKQGQNGK<br>NQIDRYYETCIGKDKGKSVSEKVDALTKIITGMNYDQFDKKRSVIEDTGRENAEREKF<br>KKIISLYLTVIYHILKNIVNINARYVIGFHCVERDAQLYKEKGYDINLKKLEEKGFSSVT<br>KLCAGIDETAPDKRKDVEKEMAERAKESIDSLESANPKLYANYIKYSDEKKAEEFTRQ<br>INREKAKTALNAYLRNTKWNVIIREDLLRIDNKTCTLFRNKAVHLEVARYVHAYINDI<br>AEVNSYFQLYHYIMQRIIMNERYEKSSGKVSEYFDAVNDEKKYNDRLLKLLCVPFGY<br>CIPRFKNLSIEALFDRNEAAKFDKEKKKVSGNS*
EsCas13d	MGKKIHARDLREQRKTDRTEKFADQNKKREAERAVPKKDAAVSVKSVSSVSSKKDN<br>VTKSMAKAAGVKSVFAVGNTVYMTSFGRGNDAVLEQKIVDTSHEPLNIDDPAYQLN<br>VVTMNGYSVTGHRGETVSAVTDNPLRRFNGRKKDEPEQSVPTDMLCLKPTLEKKFFG<br>KEFDDNIHIQLIYNILDIEKILAVYSTNAIYALNNMSADENIENSDFFMKRTTDETFDDFE<br>KKKESTNSREKADFDAFEKFIGNYRLAYFADAFYVNKKNPKGKAKNVLREDKELYSV<br>LTLIGKLRHWCVHSEEGRAEFWLYKLDELKDDFKNVLDVVYNRPVEEINNRFIENNK<br>VNIQILGSVYKNTDIAELVRSYYEFLITKKYKNMGFSIKKLRESMLEGKGYADKEYDS<br>VRNKLYQMTDFILYTGYINEDSDRADDLVNTLRSSLKEDDKTTVYCKEADYLWKKY<br>RESIREVADALDGDNIKKLSKSNIEIQEDKLRKCFISYADSVSEFTKLIYLLTRFLSGKEI<br>NDLVTTLINKFDNIRSFLEIMDELGLDRTFTAEYSFFEGSTKYLAELVELNSFVKSCSFDI<br>NAKRTMYRDALDILGIESDKTEEDIEKMIDNILQIDANGDKKLKKNNGLRNFIASNVID<br>SNRFKYLVRYGNPKKIRETAKCKPAVRFVLNEIPDAQIERYYEACCPKNTALCSANKR<br>REKLADMIAEIKFENFSDAGNYQKANVTSRTSEAEIKRKNQAIIRLYLTVMYIMLKNLV<br>NVNARYVIAFHCVERDTKLYAESGLEVGNIEKNKTNLTMAVMGVKLENGIIKTEFDK<br>SFAENAANRYLRNARWYKLILDNLKKSERAVVNEFRNTVCHLNAIRNININIKEIKEVE<br>NYFALYHYLIQKHLENRFADKKVERDTGDFISKLEEHKTYCKDFVKAYCTPFGYNLV<br>RYKNLTIDGLFDKNYPGKDDSDEQK*

RspCas13d	MAKKNKMKPRELREAQKKARQLKAAEINNNAAPAIAAMPAAEVIAPVAEKKKSSVK<br>AAGMKSILVSKNKMYITSFGKGNSAVLEYEVDNNDYNQTQLSSKGSSNIELRGVNEV<br>NITFSSKHGFESGVEINTSNPTHRSGESSPVRGDMLGLKSELEKRFFGKTFDDNIHIQLIY<br>NILDIEKILAVYVTNIVYALNNMLSIKDSESYDDFMGYLSARNTYEVFTHPDKSNLSDK<br>AKGNIKKSFSTFNDLLKTKRLGYFGLEEPKTKDTRVSQAYKKRVYHMLAIVGQIRQSV<br>FHDKSSKLDEDLYSFIDIIDSEYRETLDYLVDERFDSINKGFIQGNKVNISLLIDMMKGY<br>EADDIIRLYYDFIVLKSQKNLGFSIKKLREKMLDEYGFRFKDKQYDSVRSKMYKLMDF<br>LLFCNYYRNDVVAGEALVRKLRFSMTDDEKEGIYADEASKLWGKFRNDFENIADHM<br>NGDVIKELGKADMDFDEKILDSEKKNASDLLYFSKMIYMLTYFLDGKEINDLLTTLISK<br>FDNIKEFLKIMKSSAVDVECELTAGYKLFNDSQRITNELFIVKNIASMRKPASSAKLTM<br>FRDALTILGIDDNITDDRISEILKLKEKGKGIHGLRNFITNNVIESSRFVYLIKYANAQKIR<br>KVAKNEKVVMFVLGGIPDTQIERYYKSCVEFPDMNSSLEVKRSELARMIKNISFDDFK<br>NVKQQAKGRENVAKERAKAVIGLYLTVMYLLVKNLVNVNARYVIAIHCLERDFGLY<br>KEIIPELASKNLKNDYRILSQTLCELCDKSPNLFLKKNERLRKCVEVDINNADSSMTRK<br>YRNCIAHLTVVRELKEYIGDIRTVDSYFSIYHYVMQRCITKRENDTKQEEKIKYEDDLL<br>KNHGYTKDFVKALNSPFGYNIPRFKNLSIEQLFDRNEYLTEK*
mini-RfxCas13d	MIEKKKSFAKGMGVKSTLVSGSKVYMTTFAEGSDAFSHPKGYAVVANNPLYTGPVQ<br>QDMLGLKETLEKRYFGESADGNDNICIQVIHNILDIEKILAEYITNAAYAVNNISGLDEG<br>RNYIINYGNECYDILALLSGLRHWVVHNNEEESRISRTWLYNLDKNLDNEYISTLNYL<br>YDRITNELTNSFSKNSAEQYFRFSIMKEQKNLGFNITKLREVMLDRKDMSEIRKNHKV<br>FDSIRTKVYTMMDFVIYRYYIEEEKDIFVINLRGSFNDDQKDALYYDEANRIWRKLENI<br>MHNIKEFRGNKTREYKKKDAPRLPRILPAGRDVSAFSKLMYALTMFLDGKEINDLLTT<br>LINKFDNIQSFLKVMPLIGVNAKFVEEYAFFKDSAKIADELRLIKSFARMGEPIADARRA<br>MYIDAIRILGTKLKKGKHGMRNFIINNVISNKRFHYLIRYGDPAHLHEIAKNEAVVKFV<br>LGRIADIQKKQGQNGKNQIDRYYETCIQFDKKRSVIEDTGRENAEREKFKKIISLYLTVI<br>YHILKNIVNINARYVIGFHCVERDAQLYKEKGYDINLKKLEEQINREKAKTALNAYLR<br>NTKWNVIIREDLLRIDNKTCTLFRNKAVHLEVARYVHAYINDINDEKKYNDRLLKLLC<br>VPFGYCIPRFKNLSIEALFDRNEAAKFDKEKKKVSGNS*
mini-EsCas13d	MGKKSMAKAAGVKSVFAVGNTVYMTSFGRGNDRGETVSAVTDNPLRRFNGRKKDE<br>PEQSVPTDMLCLKPTLEKKFFGKEFDDNIHIQLIYNILDIEKILAVYSTNAIYALNNMSA<br>DENIENSDFFMKRTTDETFDDYRLAYFADAFYVNKKNPKGKAKNVLREDKELYSVLT<br>LIGKLRHWCVHSEEGRAEFWLYKLDELKDDFKNVLDVVYNRPVEEINNRFIENSYYEF<br>LITKKYKNMGFSIKKLRESMLEGKGYADKEYDSVRNKLYQMTDFILYTGYINEDSDR<br>ADDLVNTLRSSLKEDDKTTVYCKEADYLWKKYRESIREVADALDGDNIKKLSKSNIEI<br>QEDKLRKCFISYADSVSEFTKLIYLLTRFLSGKEINDLVTTLINKFDNIRSFLEIMDELGL<br>DRTFTAEYSFFEGSTKYLAELVELNSFVKSCSFDINAKRTMYRDALDILGIESDKLRNFI<br>ASNVIDSNRFKYLVRYGNPKKIRETAKCKPAVRFVLNEIPDAQIERYYEACCPKNTALC<br>KFENFSDAGNYQKANVTSRTSEAEIKRKNQAIIRLYLTVMYIMLKNLVNVNARYVIAF<br>HCNRYLRNARWYKLILDNLKKSERAVVNEFRNTVCHLNAIRNININIKEIKEVETYCK<br>DFVKAYCTPFGYNLVRYKNLTIDGLFDKNYPGKDDSDEQK*

mini-RspCas13d	MAKKSSVKAAGMKSILVSKNKMYITSFGKGNSGFESGVEINTSNPTHRSGESSPVRGD<br>MLGLKSELEKRFFGKTFDDNIHIQLIYNILDIEKILAVYVTNIVYALNNMLSIKDSESYD<br>DFMGYLSARNTKRLGYFGLEEPKTKDTRVSQAYKKRVYHMLAIVGQIRQSVFHDKSS<br>KLDEDLYSFIDIIDSEYRETLDYLVDERFDSINKGFIQGNYYDFIVLKSQKNLGFSIKKLR<br>EKMLDEYGFRFKDKQYDSVRSKMYKLMDFLLFCNYYRNDVVAGEALVRKLRFSMT<br>DDEKEGIYADEASKLWGKFRNDFENIADHMNGDVIKELGKADMDFDEKILDSEKKNA<br>SDLLYFSKMIYMLTYFLDGKEINDLLTTLISKFDNIKEFLKIMKSSAVDVECELTAGYK<br>LFNDSQRITNELFIVKNIASMRKPASSAKLTMFRDALTILGIDDNITIHGLRNFITNNVIES<br>SRFVYLIKYANAQKIRKVAKNEKVVMFVLGGIPDTQIERYYKSCVEFPDMNSSFDDFK<br>NVKQQAKGRENVAKERAKAVIGLYLTVMYLLVKNLVNVNARYVIAIHCLERDFGLY<br>KEIIPKSPNLFLKKNERLRKCVEVDINNADSSMTRKYRNCIAHLTVVRELKEYIGDIRT<br>VDSYTKDFVKALNSPFGYNIPRFKNLSIEQLFDRNEYLTEK*
PbuCas13b	MQKQDKLFVDRKKNAIFAFPKYITIMENKEKPEPIYYELTDKHFWAAFLNLARHNVYT<br>TVNHINKLLEIAELKNDEDVLNIKDSWNKQAEKLDKKVRLRDLIMKYFPFLEAAAYEI<br>TNSKSPNNKEQREKEQSEALSLNNLKNVLFIFLEKLQVLRNYYSHYKYSEEFPKPIFETS<br>LLKNMYKVFDANVRLVKRDYMHHENIDMQRDFTHLNRKKQVGRTKNIIDSPNFHYH<br>FADKEGNMTIAGLLFFVSLFLDKKDAIWMQKKLKGFKDGRNLREQMTNEVFCRSRIS<br>LPKLKMETVQTREWMQLDMLNELIRCPKSLYERLREEDRESFKVPFDIFSDDYNAEEE<br>PFKNTLVRHQDRFPYFVLRYFDLNEIFEQLRFQIDLGTYHFSIYNKLIGDEDEVRHLTHH<br>LYGFARIQDFAPQNQPEEWRKLVKDLDHFETSQKPYISKTTPHYHLENEKIGIKFCSAH<br>NNLFPSLQTDKTCNGRSKFNLGTQFTAEAFLSVHELLPMMFYYLLLTKDYSRKESADK<br>VEGIIRKEISNIYAIYDAFANGEINSIADLTRRLQNTNILQGHLPKQMISILEGRQKDMEK<br>KAERKIGEMIDDTQRRLDSLCKQTNQKIRIGKRNAGLLKSGKIADWLVNDMMRFQPV<br>QKDQNNIPINNSKANSTEYRMLQHALALFGSESSRLKAYFRQMNLVGNANPHPFLAET<br>QWEHQTNILSFYRNYLEARKKYLKGLKPQNRKQYQHFLILKVQKTNRNTLVTGWKN<br>SFNLPRGIFTQPIREWFEKHNNSKRIYDQILSFDRVGFVAKAIPLYFAEEYKDNVQPFYD<br>YPFNIGNKLKPQKGQFLDKKERVELWQKNKELFKNYPSEKKKTDLAYLDFLSWKKFE<br>RELRLIKNQDIVTWLMFKELFKTTTVEGLKIGEIHLRDIDTNTANEESNNILNRIMPMKL<br>PVKTYETDNKGNILKERPLATFYIEETETKVLKQGNFKVLAKDRRLNGLLSFAETTDID<br>LEKNPITKLSVDHELIKYQTTRISIFEMTLGLEKKLIDKYSTLPTDSFRNMLERWLQCKA<br>NRPELKNYVNSLIAVRNAFSHNQYPMYDATLFAEVKKFTLFPSVDTKKIELNIAPQLLE<br>IVGKAIKEIEKSENKN*

PspCas13b	MNIPALVENQKKYFGTYSVMAMLNAQTVLDHIQKVADIEGEQNENNENLWFHPVMS<br>HLYNAKNGYDKQPEKTMFIIERLQSYFPFLKIMAKNQREYSNGKYKQNRVEVNSNDIF<br>EVLKRAFGVLKMYRDLTNHYKTYEEKLNDGCEFLTSTEQPLSGMINNYYTVALRNM<br>NERYGYKTEDLAFIQDKRFKFVKDAYGKKKSQVNTGFFLSLQDYNGDTQKKLHLSG<br>VGIALLICLFLDKQYISIFLSRLPIFSSYNAQSEERRIIIRSFGINSIKLPKDRIHSEKSNKSV<br>AMDMLNEVKRCPDELFTTLSAEKQSRFRIISDDHNEVLMKRSSDRFVPLLLQYIDYGK<br>LFDHIRFHVNMGKLRYLLKADKTCIDGQTRVRVIEQPLNGFGRLEEAETMRKQENGTF<br>GNSGIRIRDFENMKRDDANPANYPYIVDTYTHYILENNKVEMFINDKEDSAPLLPVIED<br>DRYVVKTIPSCRMSTLEIPAMAFHMFLFGSKKTEKLIVDVHNRYKRLFQAMQKEEVT<br>AENIASFGIAESDLPQKILDLISGNAHGKDVDAFIRLTVDDMLTDTERRIKRFKDDRKSI<br>RSADNKMGKRGFKQISTGKLADFLAKDIVLFQPSVNDGENKITGLNYRIMQSAIAVYD<br>SGDDYEAKQQFKLMFEKARLIGKGTTEPHPFLYKVFARSIPANAVEFYERYLIERKFYL<br>TGLSNEIKKGNRVDVPFIRRDQNKWKTPAMKTLGRIYSEDLPVELPRQMFDNEIKSHL<br>KSLPQMEGIDFNNANVTYLIAEYMKRVLDDDFQTFYQWNRNYRYMDMLKGEYDRK<br>GSLQHCFTSVEEREGLWKERASRTERYRKQASNKIRSNRQMRNASSEEIETILDKRLSN<br>SRNEYQKSEKVIRRYRVQDALLFLLAKKTLTELADFDGERFKLKEIMPDAEKGILSEIM<br>PMSFTFEKGGKKYTITSEGMKLKNYGDFFVLASDKRIGNLLELVGSDIVSKEDIMEEFN<br>KYDQCRPEISSIVFNLEKWAFDTYPELSARVDREEKVDFKSILKILLNNKNINKEQSDIL<br>RKIRNAFDHNNYPDKGVVEIKALPEIAMSIKKAFGEYAIMK*
mini-PbuCas13b	MQKQDKLFVDRKKNAIFAFPKYITIMENKEKPEPIYYELTDKHFWAAFLNLARHNVYT<br>TINHINRRLEQAKKLDKKVRLRDLIMKHFPFLEAAAYEMTNKEQREKEQSEALSLNNL<br>KNVLFIFLEKLQVLRNYYSHYKYSEESPKPIFETSLLKNMYKVFDANVRLVKRDYMH<br>HENIDMQRDFTHFADKEGNMTIAGLLFFVSLFLDKCRSRISLPKLKLENVQTKDWMQL<br>DMLNELVRCPKSLYERLREKDRESFKVPFKNTLVRHQDRFPYFVLRYFDLNEIFEQLRF<br>QIDLGTYHFSIYNKRIGDEDEVRHLTHHLYGFARIQDFTAPHYHLENEKIGIKFCSAHN<br>NLFPSLQTDKTCNGRSKFNLGTQFTAEAFLSVHELLPMMFYYLLLTKDYSRKESADKV<br>EGIIRKEISNIILQGHLPKQMISILKGRQKDMGKEAERKIGEMIDDTQRRLDLLCKQTNQ<br>KIRIGKRNAGLLKSGKIADWLVNDMMRFQPVQKDQNNIPINNSKANSTEYRMLQRAL<br>ALFGSEILSFYRNYLEARKKYLKGLKPQNWKQYQHFLILKVQKTNRNTLVTGWKNSF<br>NLPRGIFTQILSFDRVGFVAKAIPLYFAEEYKDNVQPFYDYPFNIGNRLKPKKRQFLDK<br>KERVELWQKNKELFKNYPSEKKKTDLAYLDFLSWKKFERELRLIKNQDIVTWLMFKE<br>SNNILNRIMPMKLPVKTYETDNKGNILKERPLATFYIEETETKVLKQGNFKALVKDRR<br>LNGLFSFAISKLSVDLELIKYQTTRISIFEMTLGLEKKLIDKYSTLPTDSFRNMLERWLQ<br>CKANRPELKNYVNSLIAVRNAFSHNQYP*

mini-PspCas13b	MENQKKYFGTYSVMAMLNAQTVLDHIQKVADIEGEQPEKTMFIIERLQSYFPFLKIMA<br>KNQREYSNGKYKQNRVEVNSNDIFEVLKRAFGVLKMYRDLTNHYKTYEEKLNDGCE<br>FLTSTEQPLSGMINNYYTVALRNMNERYGYKTEDLAFIQDKLQDYNGDTQKKLHLSG<br>VGIALLICLFLDKGINSIKLPKDRIHSEKSNKSVAMDMLNEVKRCPDELFTTLSAEKQSR<br>SSDRFVPLLLQYIDYGKLFDHIRFHVNMGKLRYLLKADKTCIDGQTRVRVIEQPLNGF<br>GRLEEAYTHYILENNKVEMFINDKEDSAPLLPVIEDDRYVVKTIPSCRMSTLEIPAMAF<br>HMFLFGSKKTEKLIVDVHNRYKRLFQAMQKEESDLPQKILDLISGNAHGKDVDAFIRL<br>TVDDMLTDTERRIKRFKDDRKSIRSADNKMGKRGFKQISTGKLADFLAKDIVLFQPSV<br>NDGENKITGLNYRIMQSAIAVYDSGDDYEAKAVEFYERYLIERKFYLTGLSNEIKKGN<br>RVDVPFIRRDQNKWKTPAMKTLGRIYSEDLPVELPRQMFDFNNANVTYLIAEYMKRV<br>LDDDFQTFYQWNRNYRYMDMLKGEYDRKGSLQHCFTSVEEREGLWKERASRTERY<br>RKQASNKIRSNRQMRNASSEEIETILDKRLSNSRNEYQKSEKVIRRYRVQDALLFLLAK<br>DAEKGILSEIMPMSFTFEKGGKKYTITSEGMKLKNYGDFFVLASDKRIGNLLELVGSDI<br>VSKEDIMEEFNKYDQCRPEISSIVFNLEKWAFDTYPELSARVDREEKVDFKSILKILLNN<br>KNINKEQSDILRKIRNAFDHNNYP*
REPAIRVx	MIEKKKSFAKGMGVKSTLVSGSKVYMTTFAEGSDARLEKIVEGDSIRSVNEGEAFSAE<br>MADKNAGYKIGNAKFSHPKGYAVVANNPLYTGPVQQDMLGLKETLEKRYFGESADG<br>NDNICIQVIHNILDIEKILAEYITNAAYAVNNISGLDKDIIGFGKFSTVYTYDEFKDPEHH<br>RAAFNNNDKLINAIKAQYDEFDNFLDNPRLGYFGQAFFSKEGRNYIINYGNECYDILAL<br>LSGLAHWVVANNEEESRISRTWLYNLDKNLDNEYISTLNYLYDRITNELTNSFSKNSA<br>ANVNYIAETLGINPAEFAEQYFRFSIMKEQKNLGFNITKLREVMLDRKDMSEIRKNHK<br>VFDSIRTKVYTMMDFVIYRYYIEEDAKVAAANKSLPDNEKSLSEKDIFVINLRGSFNDD<br>QKDALYYDEANRIWRKLENIMHNIKEFRGNKTREYKKKDAPRLPRILPAGRDVSAFSK<br>LMYALTMFLDGKEINDLLTTLINKFDNIQSFLKVMPLIGVNAKFVEEYAFFKDSAKIAD<br>ELRLIKSFARMGEPIADARRAMYIDAIRILGTNGSKRTADGSEFESPKKKRKVGSQLHL<br>PQVLADAVSRLVLGKFGDLTDNFSSPHARRKVLAGVVMTTGTDVKDAKVISVSTGTK<br>CINGEYMSDRGLALNDCHAEIISRRSLLRFLYTQLELYLNNKDDQKRSIFQKSERGGFR<br>LKENVQFHLYISTSPCGDARIFSPHEPILEEPADRHPNRKARGQLRTKIESGQGTIPVRSN<br>ASIQTWDGVLQGERLLTMSCSDKIARWNVVGIQGSLLSIFVEPIYFSSIILGSLYHGDHL<br>SRAMYQRISNIEDLPPLYTLNKPLLSGISNAEARQPGKAPNFSVNWTVGDSAIEVINATT<br>GKDELGRASRLCKHALYCRWMRVHGKVPSHLLRSKITKPNVYHESKLAAKEYQAAK<br>ARLFTAFIKAGLGAWVEKPTEQDQFSLTGSKRTADGSEFESPKKKRKVGSMRNFIINN<br>VISNKRFHYLIRYGDPAHLHEIAKNEAVVKFVLGRIADIQKKQGQNGKNQIDRYYETCI<br>GKDKGKSVSEKVDALTKIITGMNYDQFDKKRSVIEDTGRENAEREKFKKIISLYLTVIY<br>HILKNIVNINARYVIGFHCVERDAQLYKEKGYDINLKKLEEKGFSSVTKLCAGIDETAP<br>DKRKDVEKEMAERAKESIDSLESANPKLYANYIKYSDEKKAEEFTRQINREKAKTALN<br>AYLRNTKWNVIIREDLLRIDNKTCTLFANKAVALEVARYVHAYINDIAEVNSYFQLYH<br>YIMQRIIMNERYEKSSGKVSEYFDAVNDEKKYNDRLLKLLCVPFGYCIPRFKNLSIEAL<br>FDRNEAAKFDKEKKKVSGNS*

mini-<br>REPAIRVx	MIEKKKSFAKGMGVKSTLVSGSKVYMTTFAEGSDAFSHPKGYAVVANNPLYTGPVQ<br>QDMLGLKETLEKRYFGESADGNDNICIQVIHNILDIEKILAEYITNAAYAVNNISGLDEG<br>RNYIINYGNECYDILALLSGLAHWVVANNEEESRISRTWLYNLDKNLDNEYISTLNYL<br>YDRITNELTNSFSKNSAEQYFRFSIMKEQKNLGFNITKLREVMLDRKDMSEIRKNHKV<br>FDSIRTKVYTMMDFVIYRYYIEEEKDIFVINLRGSFNDDQKDALYYDEANRIWRKLENI<br>MHNIKEFRGNKTREYKKKDAPRLPRILPAGRDVSAFSKLMYALTMFLDGKEINDLLTT<br>LINKFDNIQSFLKVMPLIGVNAKFVEEYAFFKDSAKIADELRLIKSFARMGEPIADARRA<br>MYIDAIRILGTGSKRTADGSEFESPKKKRKVGSQLHLPQVLADAVSRLVLGKFGDLTD<br>NFSSPHARRKVLAGVVMTTGTDVKDAKVISVSTGTKCINGEYMSDRGLALNDCHAEII<br>SRRSLLRFLYTQLELYLNNKDDQKRSIFQKSERGGFRLKENVQFHLYISTSPCGDARIFS<br>PHEPILEEPADRHPNRKARGQLRTKIESGQGTIPVRSNASIQTWDGVLQGERLLTMSCS<br>DKIARWNVVGIQGSLLSIFVEPIYFSSIILGSLYHGDHLSRAMYQRISNIEDLPPLYTLNK<br>PLLSGISNAEARQPGKAPNFSVNWTVGDSAIEVINATTGKDELGRASRLCKHALYCRW<br>MRVHGKVPSHLLRSKITKPNVYHESKLAAKEYQAAKARLFTAFIKAGLGAWVEKPTE<br>QDQFSLTGSKRTADGSEFESPKKKRKVGSMRNFIINNVISNKRFHYLIRYGDPAHLHEI<br>AKNEAVVKFVLGRIADIQKKQGQNGKNQIDRYYETCIQFDKKRSVIEDTGRENAEREK<br>FKKIISLYLTVIYHILKNIVNINARYVIGFHCVERDAQLYKEKGYDINLKKLEEQINREK<br>AKTALNAYLRNTKWNVIIREDLLRIDNKTCTLFANKAVALEVARYVHAYINDINDEK<br>KYNDRLLKLLCVPFGYCIPRFKNLSIEALFDRNEAAKFDKEKKKVSGNS*

Supplementary Table 2. Primers used in this study.

Primers for cloning Δ1, Δ2, Δ3 RfxCas13d expression plasmids

Name	Sequence
Δ1 RfxCas13d	Frament-F:gaaggcagcgacgccttcagccatcctaagggct
	Frament-R:cgtatcgtagttatctacacgacgggg
	Vector-F:gataactacgatacgggagggctt
	Vector-R:ggcgtcgctgccttcg
Δ2 RfxCas13d	Frament-F:atctccggcctggatgagggcagaaattacatcatcaattacggcaacga
	Frament-R:cgtatcgtagttatctacacgacgggg
	Vector-F:gataactacgatacgggagggctt
	Vector-R:atccaggccggagatattgttgac
Δ3 RfxCas13d	Frament-F:ttctccaagaactccgccgaacaatatttcagattcagc
	Frament-R:cctgtagcaatggcaacaacgttgcgc
	Vector-F:tgccattgctacaggcatcgt
	Vector-R:ggagttcttggagaaggagttggt

Primers for cloning mammalian expression plasmids

Name	Sequence
EsCas13d	Frament-F:cttaagcttgccaccatgaaaatcgaagaaggtaaaggtcacc
	Frament-R:gactcgagcggccgcttatttctgctcgtcgctatcatctttacccgg
	Vector-F:gcggccgctcgagtc
	Vector-R:ggtggcaagcttaagtttaaacgct
RspCas13d	Frament-F:cttaagcttgccaccatgaaaatcgaagaaggtaaaggtcacc
	Frament-R:gactcgagcggccgcttatttttccgtcagatattcattacgatcaaacagctgctcg
	Vector-F:gcggccgctcgagtc
	Vector-R:ggtggcaagcttaagtttaaacgct
PspCas13b	Frament-F:cttaagcttgccaccatgaacatccccg
	Frament-R:gactcgagcggccgccttcatgatggcgtactcccc
	Vector-F:gcggccgctcgagtc
	Vector-R:ggtggcaagcttaagtttaaacgct

QPCR primers used in the study

STAT6 -qPCR-F	GAGGATCCAGATTTGGAAACGG
STAT6 -qPCR-R	AATGTCCACCAGGCTTTCAC
NRAS -qPCR-F	CACTGACAATCCAGCTAATCCA
NRAS -qPCR-R	TCTGCTCCCTGTAGAGGTTAATA
LMNA -qPCR-F	CTCCTTGGGCACAGAACC
LMNA -qPCR-R	TGGGCTGTCTAGGACTCAG
EGFR -qPCR-F	AAGTGTGATCCAAGCTGTCC

EGFR -qPCR-R	TGCTGGGCACAGATGATTT
KRAS -qPCR-F	GCCTTCTAGAACAGTAGACACAA
KRAS -qPCR-R	TCCACTCTCTGTCTTGTCTTTG
MATAL1 -qPCR-F	AACTTGTAGACTGGAGAAGATAGG
MATAL1 -qPCR-R	GAGATCAGCTTCCGCTAAGAT
HRAS -qPCR-F	GGAAGCAGGTGGTCATTGAT
HRAS -qPCR-R	GGAAGCAGGTGGTCATTGAT
NF2 -qPCR-F	AAGATCTACTGCCCTCCTGA
NF2 -qPCR-R	GGGTCGTAGTCACCATACTTG
STAT1 -qPCR-F	GGAATCTGTCCTTCTTCCTGAC
STAT1 -qPCR-R	GGTCCACATTGAGACCTCTTT
STAT1 -2-qPCR-F	TCTCAATGTGGACCAGCTGAACA
STAT1 -2-qPCR-R	GGTCCACATTGAGACCTCTTT
RAF1 -qPCR-F	GGCTCAGGGAATGGACTATTT
RAF1 -qPCR-R	GAGTAGACATCCGACTGGAAAC
TRAF7 -qPCR-F	TCTGGTGGCAGCGTCTA
TRAF7 -qPCR-R	TTGGACTCAATGTCCCACAC
PPARG -qPCR-F	GTCGGTTTCAGAAATGCCTTG
PPARG -qPCR-R	ATCTCCGCCAACAGCTTC
XYL2 -qPCR-F	AACTTCGAGCAGGTGATTGT
XYL2 -qPCR-R	CAATTAGGCAGCCCTCATCA
RPASP58 -qPCR-F	CCCTGCTGATGTCAGTGTTAT
RPASP58 -qPCR-R	GTGAAGGTTCCAGGAGTGAAG
NFKB1 -qPCR-F	GCTACTCTGGCGCAGAAATTA
NFKB1 -qPCR-R	CAGAGACCTCATAGTTGTCCATAAG
PPIB -qPCR-F	GAGATGGCACAGGAGGAAAG
PPIB -qPCR-R	ACTGTCGTGATGAAGAACTGG
B4GALNT1 -qPCR-F	GACCAGCTGCTCATAGCC
B4GALNT1 -qPCR-R	GACCAGAAGCTGCCTGAA

Primers for RNA editing

COG3 -F	CAGATGCATAGATAGGGCAGTGTTCCAAGGA
COG3 -R	ACCTTTGTCATGAACTCCTCCAGCTGTTC
GLI2 -F	CAGAACTTTGATCCTTACCTC
GLI2 -R	CCCATATCCCAGAGTATCAGTAGGTGG
PPIB -F	CTGCTGCCGGGACCTTCTG
PPIB -R	CTCCGCCCTGGATCATGAAGT

Primers for Deep sequence of RNA editing

COG3 -F	GGAGTGAGTACGGTGTGCGGGCAGTGTTCCAAGGATTA

COG3 -R	GAGTTGGATGCTGGATGGGACAGTCATAGGGTTCAGGATTT
GLI2 -F	GGAGTGAGTACGGTGTGCAGCTAGAGTCCAGAGGTTCA
GLI2 -R	GAGTTGGATGCTGGATGGAGAGTATCAGTAGGTGGGAAGT

Primers for *Pcsk9* mRNA knockdown

Pcsk9 -qPCR-F	ACAGGCGAGCAAGTGTG
Pcsk9 -qPCR-R	CTGTGCCCTTCCCTTGAC

Primers for Detecting mini-RfxCas13d expression

mini-RfxCas13d-primer1-F	CTGGCTCTACAACCTCGATAAG
mini-RfxCas13d-primer1-R	GGGCGTCCTTCTTCTTATACTC
mini-RfxCas13d-primer2-F	GGATCGCTGACATCCAGAAA
mini-RfxCas13d-primer2-R	TTGGCCTTCTCCCTGTTAATC

Supplementary Table 3. CrRNA spacer sequences for RNA knockdown experiments.

Name	Spacer sequence	DR sequence	Interference Mechanism	First figure
STAT6	ACACCTCTCCTGGAGTGGGGCCA	AACCCCTACCAACTGGTCGGGGTTTGAAAC	RfxCas13d	Fig 2b
NRAS	GAAGGCTTCCTCTGTGTATTTGC			Fig 2b
LMNA	ACCGTGACACTGGAGGCAGAAGA			Fig 2b
EGFR	GGGGTGCAGGAGAGGAGAACTGCCAGAAAC			Fig 2b,2c
KRAS	CAGGCTCAGGACTTAGCAAGAAGTTATGGA			Fig 2c
MATAL1	CTGATCTCCAATGCTCTTCAGTA			Fig 2c
HRAS	GTCCGAGTCCTTCACCCGTTTGATCTGCTCC			Fig 2c
NF2	TAGTCACCATACTTGGCCTGGACGGCGTAA			Fig 2c
STAT1	GCGGAACCCAGGAATCTGTCCTT			Fig 2c
STAT1-2	TCTCAATGTGGACCAGCTGAACATGTTGGG			Fig 2c
RAF1	GGACAGCAGGATGATTGAGGATGCAATTCG			Fig 2c
TRAF7	ACAGCAATGGAGTAGACGCTGCC			Fig 2c
PPARG	AATGCCTTGCAGTGGGGATGTCTCATAATG			Fig 2c
XYL2	GGAGCCGGCACTGATGAGGGCTGCCTAATT			Fig 2c
RPASP58	CCAGTATTCCTGGAGGATATAAC			Fig 2c
NFKB1	ACTTATGGACAACTATGAGGTCTCTGGGGG			Fig 2c
EGFR	GGGGTGCAGGAGAGGAGAACTGCCAGAAAC	CACCCGTGCAAAATTGCAGGGGTCTAAAAC	EsCas13d	Fig 3e
KRAS	CAGGCTCAGGACTTAGCAAGAAGTTATGGA			Fig 3e
MATAL1	CTGATCTCCAATGCTCTTCAGTAGGGTCAT			Fig 3e
PPIB	CAAACTGAAGCACTACGGGCCTGGCTGGGT			Fig 3e
NRAS	CTCAGCCAAGACCAGACAGGGTGTTGAAGA			Fig 3e
PPARG	AATGCCTTGCAGTGGGGATGTCTCATAATG			Fig 3e
EGFR	GGGGTGCAGGAGAGGAGAACTGCCAGAAAC	CACTGGTGCAAATTTGCACTAGTCTAAAAC	RspCas13d	Fig 3f
KRAS	CAGGCTCAGGACTTAGCAAGAAGTTATGGA			Fig 3f
MATAL1	CTGATCTCCAATGCTCTTCAGTAGGGTCAT			Fig 3f
PPIB	CAAACTGAAGCACTACGGGCCTGGCTGGGT			Fig 3f
NRAS	CTCAGCCAAGACCAGACAGGGTGTTGAAGA			Fig 3f
PPARG	AATGCCTTGCAGTGGGGATGTCTCATAATG			Fig 3f
EGFR	GGGGTGCAGGAGAGGAGAACTGCCAGAAAC	GTTGCATCTGCCTTCTTTTTGAAAGGTAAAAACAAC	PbuCas13b	Fig 5e
KRAS	CAGGCTCAGGACTTAGCAAGAAGTTATGGA			Fig 5e
MATAL1	CTGATCTCCAATGCTCTTCAGTAGGGTCAT			Fig 5e
PPIB	CAAACTGAAGCACTACGGGCCTGGCTGGGT			Fig 5e
NRAS	CTCAGCCAAGACCAGACAGGGTGTTGAAGA			Fig 5e
PPARG	AATGCCTTGCAGTGGGGATGTCTCATAATG			Fig 5e
HRAS	GGAGCAGATCAAACGGGTGAAGGACTCGGA			Fig 5e
B4GALNT1	TGAGCCTTCAGGCAGCTTCTGGTCAGGAGG			Fig 5e
EGFR	GGGGTGCAGGAGAGGAGAACTGCCAGAAAC	GTTGTGGAAGGTCCAGTTTTGAGGGGCTATTACAAC	PspCas13b	Fig 5f
KRAS	CAGGCTCAGGACTTAGCAAGAAGTTATGGA			Fig 5f
MATAL1	CTGATCTCCAATGCTCTTCAGTAGGGTCAT			Fig 5f
PPIB	CAAACTGAAGCACTACGGGCCTGGCTGGGT			Fig 5f
NRAS	CTCAGCCAAGACCAGACAGGGTGTTGAAGA			Fig 5f
PPARG	AATGCCTTGCAGTGGGGATGTCTCATAATG			Fig 5f
HRAS	GGAGCAGATCAAACGGGTGAAGGACTCGGA			Fig 5f
B4GALNT1	TGAGCCTTCAGGCAGCTTCTGGTCAGGAGG			Fig 5f

Supplementary Table 4. CrRNA sequences used in this study for RNA editing in mammalian cells.

Editing site	Spacer sequence	Mismatch distance	Editing system	First figure
COG3	GCTCCATTTCACACTGAATTCACCATTAAGGAAATTTCCCTGGACCTCAA	17/50	REPAIRVx	Fig 6b
GLI2	CCCAGCATCACTGAGAATGCTGCCATGGATGCTAGAGGGCTACAGGAAGA	17/50		Fig 6b
PPIB	GTATTTTGACCTACGAATTGGAGATGAAGATGTAGGCCGGGTGATCTTTG	17/50		Fig 6b
COG3	GCTCCATTTCACACTGAATTCACCATTAAGGAAATTTCCCTGGACCTCAA	17/50	mini-REPAIRVx	Fig 6b
GLI2	CCCAGCATCACTGAGAATGCTGCCATGGATGCTAGAGGGCTACAGGAAGA	17/50		Fig 6b
PPIB	GTATTTTGACCTACGAATTGGAGATGAAGATGTAGGCCGGGTGATCTTTG	17/50		Fig 6b

Supplementary Table5.CrRNA spacer sequences for *Pcsk9* knockdown experiments.

Name	Spacer sequence	Interference Mechanism	First figure
Pcsk9 -1	CTTCAACAGCGTGCCGGAGGAGGATGGGAC	RfxCas13d	Fig S15a
Pcsk9- 2	GCGAGCAAGTGTGACAGCCACGGCACCCAC		Fig S15a
Pcsk9- 3	TGGCAGGTGTGGTCAGCGGCCGGGATGCTG		Fig S15a
Pcsk9- 1	CTTCAACAGCGTGCCGGAGGAGGATGGGAC	mini-RfxCas13d	Fig S15a
Pcsk9- 2	GCGAGCAAGTGTGACAGCCACGGCACCCAC		Fig S15a
Pcsk9- 3	TGGCAGGTGTGGTCAGCGGCCGGGATGCTG		Fig S15a
