# Supplementary material for: A strategy for Cas13 miniaturization based on the structure and AlphaFold
Source: Nat Commun. 2023 Sep 8;14:5545. doi: 10.1038/s41467-023-41320-8 (PMC10491665; doi:10.1038/s41467-023-41320-8)
Supplement: Supplementary file 2 — Reporting Summary [file 41467_2023_41320_MOESM2_ESM.pdf]

nature portfolio

Corresponding author(s): Tao Zhang, Liangxue Lai, Tingting Sui, Zhanjun Li

Last updated by author(s): 2023.08.22

Reporting Summary

Nature Portfolio wishes to improve the reproducibility of the work that we publish. This form provides structure for consistency and transparency in reporting. For further information on Nature Portfolio policies, see our Editorial Policies and the Editorial Policy Checklist.

Statistics

For all statistical analyses, confirm that the following items are present in the figure legend, table legend, main text, or Methods section.

n/a	Confirmed	
☐	☒	The exact sample size (*n*) for each experimental group/condition, given as a discrete number and unit of measurement
☐	☒	A statement on whether measurements were taken from distinct samples or whether the same sample was measured repeatedly
☐	☒	The statistical test(s) used AND whether they are one- or two-sided<br>*Only common tests should be described solely by name; describe more complex techniques in the Methods section.*
☒	☐	A description of all covariates tested
☐	☒	A description of any assumptions or corrections, such as tests of normality and adjustment for multiple comparisons
☐	☒	A full description of the statistical parameters including central tendency (e.g. means) or other basic estimates (e.g. regression coefficient) AND variation (e.g. standard deviation) or associated estimates of uncertainty (e.g. confidence intervals)
☒	☐	For null hypothesis testing, the test statistic (e.g. *F*, *t*, *r*) with confidence intervals, effect sizes, degrees of freedom and *P* value noted<br>*Give P values as exact values whenever suitable.*
☒	☐	For Bayesian analysis, information on the choice of priors and Markov chain Monte Carlo settings
☒	☐	For hierarchical and complex designs, identification of the appropriate level for tests and full reporting of outcomes
☒	☐	Estimates of effect sizes (e.g. Cohen's *d*, Pearson's *r*), indicating how they were calculated

Our web collection on statistics for biologists contains articles on many of the points above.

Software and code

Policy information about availability of computer code

Data collection	https://github.com/sokrypton/ColabFold/blob/main/AlphaFold2.ipynb;version:ColabFold v1.5.2
Data analysis	https://www.cgl.ucsf.edu/chimerax/;version:ChimeraX 1.3

For manuscripts utilizing custom algorithms or software that are central to the research but not yet described in published literature, software must be made available to editors and reviewers. We strongly encourage code deposition in a community repository (e.g. GitHub). See the Nature Portfolio guidelines for submitting code & software for further information.

Data

Policy information about availability of data

All manuscripts must include a data availability statement. This statement should provide the following information, where applicable:

- Accession codes, unique identifiers, or web links for publicly available datasets
- A description of any restrictions on data availability
- For clinical datasets or third party data, please ensure that the statement adheres to our policy

Source data are provided with this paper. The RNA-seq data and DNA sequences data generated in this study have been deposited in the SRA under BioProject PRJNA982412. {PRJNA1007948 Details | Manage Data | Submission Portal (nih.gov)}. The Q-PCR data generated in this study are provided in the Source Data file.

Research involving human participants, their data, or biological material

Policy information about studies with human participants or human data. See also policy information about sex, gender (identity/presentation), and sexual orientation and race, ethnicity and racism.

Reporting on sex and gender	n/a
Reporting on race, ethnicity, or other socially relevant groupings	n/a
Population characteristics	n/a
Recruitment	n/a
Ethics oversight	n/a

Note that full information on the approval of the study protocol must also be provided in the manuscript.

Field-specific reporting

Please select the one below that is the best fit for your research. If you are not sure, read the appropriate sections before making your selection.

[x] Life sciences [ ] Behavioural & social sciences [ ] Ecological, evolutionary & environmental sciences

For a reference copy of the document with all sections, see nature.com/documents/nr-reporting-summary-flat.pdf

Life sciences study design

All studies must disclose on these points even when the disclosure is negative.

Sample size	Experiments in cell lines were performed in triplicates n=3,unless otherwise noted. These sample sizes were selected based on following literature that used similar sample sizes to obtain results. eg:<br>Sui T, Song Y, Liu Z, Chen M, Deng J, Xu Y, Lai L, Li Z. CRISPR-induced exon skipping is dependent on premature termination codon mutations. Genome Biol. 2018 Oct 17;19(1):164. doi: 10.1186/s13059-018-1532-z. PMID: 30333044; PMCID: PMC6193291.
Data exclusions	No data was excluded.
Replication	We tested experimental conditions using different crRNAs to ensure robustness. We also performed at least three biological replicates for each experiment and the experimental results could be successfully reproduced.
Randomization	Due to the small sample,randomization was not relevant for this study. Covariates were controlled for by running controls in parallel whenever applicable.
Blinding	Blinding was not relevant to our study because in general, based on the prior experience of other groups in the field, these types of assays do not require blinding. The prior experience in following publications. eg:<br>Cox DBT, Gootenberg JS, Abudayyeh OO, Franklin B, Kellner MJ, Joung J, Zhang F. RNA editing with CRISPR-Cas13. Science. 2017 Nov 24;358 (6366):1019-1027. doi: 10.1126/science.aaq0180. Epub 2017 Oct 25. PMID: 29070703; PMCID: PMC5793859.<br>He B, Peng W, Huang J, Zhang H, Zhou Y, Yang X, Liu J, Li Z, Xu C, Xue M, Yang H, Huang P. Modulation of metabolic functions through Cas13d-mediated gene knockdown in liver. Protein Cell. 2020 Jul;11(7):518-524. doi: 10.1007/s13238-020-00700-2. PMID: 32185621; PMCID: PMC7095259.<br>Konermann S, Lotfy P, Brideau NJ, Oki J, Shokhirev MN, Hsu PD. Transcriptome Engineering with RNA-Targeting Type VI-D CRISPR Effectors. Cell. 2018 Apr 19;173(3):665-676.e14. doi: 10.1016/j.cell.2018.02.033. Epub 2018 Mar 15. PMID: 29551272; PMCID: PMC5910255.

Reporting for specific materials, systems and methods

We require information from authors about some types of materials, experimental systems and methods used in many studies. Here, indicate whether each material, system or method listed is relevant to your study. If you are not sure if a list item applies to your research, read the appropriate section before selecting a response.

Materials & experimental systems

n/a	Involved in the study	
	x	Antibodies
	x	Eukaryotic cell lines
x		Palaeontology and archaeology
	x	Animals and other organisms
x		Clinical data
x		Dual use research of concern
x		Plants

Methods

n/a	Involved in the study	
x		ChIP-seq
x		Flow cytometry
x		MRI-based neuroimaging

Antibodies

Antibodies used

PROTEINTECH GROUP: PCSK9 55206-1-AP ; GAPDH 10494-1-AP. MERCK:anti-HA antibody H6908. Beyotime Biotechnology: goat anti-rabbit IgG H+L A0208.

Validation

The PCSK9 antibody has been validated in following publications.eg:
Liu X, Bao X, Hu M, Chang H, Jiao M, Cheng J, Xie L, Huang Q, Li F, Li CY. Inhibition of PCSK9 potentiates immune checkpoint therapy for cancer. Nature. 2020 Dec;588(7839):693-698. doi: 10.1038/s41586-020-2911-7. Epub 2020 Nov 11. PMID: 33177715; PMCID: PMC7770056.Application: WB. Species: Human,mouse.
Li Z, Zhu H, Liu H, Liu D, Liu J, Jiang J, Zhang Y, Qin Z, Xu Y, Peng Y, Liu B, Long Y. Evolocumab loaded Bio-Liposomes for efficient atherosclerosis therapy. J Nanobiotechnology. 2023 May 19;21(1):158. doi: 10.1186/s12951-023-01904-4. PMID: 37208681; PMCID: PMC10199622.Application: WB. Species: mouse.
Wang J, Wang Y, Yang X, Lin P, Liu N, Li X, Zhang B, Guo S. Purification, structural characterization, and PCSK9 secretion inhibitory effect of the novel alkali-extracted polysaccharide from Cordyceps militaris. Int J Biol Macromol. 2021 May 15;179:407-417. doi: 10.1016/j.ijbiomac.2021.02.191. Epub 2021 Mar 1. PMID: 33662421.Application: WB. Species:mouse.

The GAPDH antibody has been validated in following publications.eg:
Wu N, Sun H, Zhao X, Zhang Y, Tan J, Qi Y, Wang Q, Ng M, Liu Z, He L, Niu X, Chen L, Liu Z, Li HB, Zeng YA, Roulis M, Liu D, Cheng J, Zhou B, Ng LG, Zou D, Ye Y, Flavell RA, Ginhoux F, Su B. MAP3K2-regulated intestinal stromal cells define a distinct stem cell niche. Nature. 2021 Apr;592(7855):606-610. doi: 10.1038/s41586-021-03283-y. Epub 2021 Mar 3. PMID: 33658717.Application: WB. Species: mouse.
Lu XY, Shi XJ, Hu A, Wang JQ, Ding Y, Jiang W, Sun M, Zhao X, Luo J, Qi W, Song BL. Feeding induces cholesterol biosynthesis via the mTORC1-USP20-HMGCR axis. Nature. 2020 Dec;588(7838):479-484. doi: 10.1038/s41586-020-2928-y. Epub 2020 Nov 11. PMID: 33177714.Application: WB. Species: Human,mouse.
Shu J, Wu C, Wu Y, Li Z, Shao S, Zhao W, Tang X, Yang H, Shen L, Zuo X, Yang W, Shi Y, Chi X, Zhang H, Gao G, Shu Y, Yuan K, He W, Tang C, Zhao Y, Deng H. Induction of pluripotency in mouse somatic cells with lineage specifiers. Cell. 2013 May 23;153(5):963-75. doi: 10.1016/j.cell.2013.05.001. Erratum in: Cell. 2015 May 21;161(5):1229. PMID: 23706735; PMCID: PMC4640445.Application: WB. Species:mouse.

The anti-HA antibody has been validated in following publications.eg:
Doron-Mandel E, Koppel I, Abraham O, Rishal I, Smith TP, Buchanan CN, Sahoo PK, Kadlec J, Oses-Prieto JA, Kawaguchi R, Alber S, Zahavi EE, Di Matteo P, Di Pizio A, Song DA, Okladnikov N, Gordon D, Ben-Dor S, Haffner-Krausz R, Coppola G, Burlingame AL, Jungwirth P, Twiss JL, Fainzilber M. The glycine arginine-rich domain of the RNA-binding protein nucleolin regulates its subcellular localization. EMBO J. 2021 Oct 18;40(20):e107158. doi: 10.15252/embj.2020107158. Epub 2021 Sep 13. PMID: 34515347; PMCID: PMC8521312.Application:WB. Species: Human.

The goat anti-rabbit IgG H+L has been validated in following publications.eg:
Huiling Li, Yangao Huo, Xi He, Liping Yao, Hao Zhang, Yiqiang Cui, Huijuan Xiao, Wenxiu Xie, Dejiu Zhang, Yue Wang, Shu Zhang, Haixia Tu, Yiwei Cheng, Yueshuai Guo, Xintao Cao, Yunfei Zhu, Tao Jiang, Xuejiang Guo, Yan Qin, Jiahao Sha. A male germ-cell-specific ribosome controls male fertility Nature. 2022 Dec;612(7941):725-731.;doi: 10.1038/s41586-022-05508-0. Application:WB. Species: Human.
Ping He, Banghui Liu, Xijie Gao, Qihong Yan, Rongjuan Pei, Jing Sun, Qiuluan Chen, Ruitian Hou, Zimu Li, Yanjun Zhang, Jincun Zhao, Hao Sun, Bo Feng, Qian Wang, Haisu Yi, Peiyu Hu, Pingchao Li, Yudi Zhang, Zhilong Chen, Xuefeng Niu, Xiaolin Zhong, Liang Jin, Xiaofeng Liu, Kun Qu, Katarzyna A Ciazynska, Andrew P Carter, John A G Briggs, Jizheng Chen, Jinsong Liu, Xinwen Chen, Jun He, Ling Chen, Xiaoli Xiong. SARS-CoV-2 Delta and Omicron variants evade population antibody response by mutations in a single spike epitope. Nat Microbiol. 2022 Oct;7(10):1635-1649.;doi: 10.1038/s41564-022-01235-4.
Cheng Wang, Shaobo Wang, Yin Chen, Jianqi Zhao, Songling Han, Gaomei Zhao, Jing Kang, Yong Liu, Liting Wang, Xiaoyang Wang, Yang Xu, Song Wang, Yi Huang, Junping Wang, Jinghong Zhao.Membrane Nanoparticles Derived from ACE2-Rich Cells Block SARS-CoV-2 Infection.ACS Nano. 2021 Apr 27;15(4):6340-6351.;doi: 10.1021/acsnano.0c06836.Application:WB. Species: Human.

Eukaryotic cell lines

Policy information about cell lines and Sex and Gender in Research

Cell line source(s)

HEK293T and N2A cells were obtained from cell bank of ATCC.

Authentication	Cell lines were authenticated by the supplier and not further authenticated after receipt.
Mycoplasma contamination	Cell lines were not tested.
Commonly misidentified lines (See ICLAC register)	None of the cell lines used was listed in the database of ICLAC.

Animals and other research organisms

Policy information about studies involving animals; ARRIVE guidelines recommended for reporting animal research, and Sex and Gender in Research

Laboratory animals	8-week-old male ICR mice;housing conditions:12 hours light/12 hours dark,23±3 °C temperature,50%~70% humidity.
Wild animals	No wild animals was used in this study.
Reporting on sex	Sex was not considered in study design.
Field-collected samples	None of the samples was collected in the field.
Ethics oversight	Institutional Animal Care and Use Committee of Jilin University,IACUC

Note that full information on the approval of the study protocol must also be provided in the manuscript.
