# Supplementary material for: A strategy for Cas13 miniaturization based on the structure and AlphaFold
Source: Nat Commun. 2023 Sep 8;14:5545. doi: 10.1038/s41467-023-41320-8 (PMC10491665; doi:10.1038/s41467-023-41320-8)
Supplement: Supplementary file 3 — Description of Additional Supplementary Files [file 41467_2023_41320_MOESM3_ESM.pdf]

Title: Supplementary Video 1:

Description: The rearrangement of NTD domain of EsCas13d during transition from binary complex (6E9E) to ternary complex(6E9F). The color of EsCas13d coded as indicated in Fig.3c. Truncated regions showed in dark gray. The interaction sites showed in red.

Title: Supplementary Video 2:

Description: The rearrangement of HEPN1 domain of EsCas13d during transition from binary complex (6E9E) to ternary complex(6E9F). The color of EsCas13d coded as indicated in Fig.3c. Truncated regions showed in dark gray. The interaction sites showed in red.

Title: Supplementary Video 3:

Description: The rearrangement of Helical1 domain of EsCas13d during transition from binary complex (6E9E) to ternary complex(6E9F). The color of EsCas13d coded as indicated in Fig.3c. Truncated regions showed in dark gray. The interaction sites showed in red.

Title: Supplementary Video 4:

Description: The rearrangement of Helical2 domain of EsCas13d during transition from binary complex (6E9E) to ternary complex(6E9F). The color of EsCas13d coded as indicated in Fig.3c. Truncated regions showed in dark gray. The interaction sites showed in red.

Title: Supplementary Video 5:

Description: The rearrangement of HEPN2 domain of EsCas13d during transition from binary complex (6E9E) to ternary complex(6E9F). The color of EsCas13d coded as indicated in Fig.3c. Truncated regions showed in dark gray. The interaction sites showed in red.
